# Initial Experience with the 4D Mini-TEE Probe in the Adult Population

**DOI:** 10.3390/jcm13216450

**Published:** 2024-10-28

**Authors:** Konstantinos Papadopoulos, Ignatios Ikonomidis, Augustin Coisne, Özge Özden Kayhan, Apostolos Tzikas, Nikolaos Fragakis, Antonios P. Antoniadis, Mani A Vannan, Erwan Donal

**Affiliations:** 1 Echocardiography Laboratory, European Interbalkan Medical Center, 57001 Thessaloniki, Greece; 2 Echocardiography Laboratory, 2nd Cardiology Department, Medical School, Attikon University Hospital, National and Kapodistrian University of Athens, 12462 Athens, Greece; ignoik@gmail.com; 3 University Lille, Inserm, CHU Lille, Institut Pasteur de Lille, U1011—European Genomic Institute for Diabetes (EGID), F-59000 Lille, France; augustincoisne@hotmail.com; 4 Cardiovascular Research Foundation, New York, NY 10019, USA; 5 Cardiology Department, Memorial Bahcelievler Hospital, 34180 Istanbul, Turkey; ozgeozdenctf@hotmail.com; 6 Cardiology Department, European Interbalkan Medical Center, 57001 Thessaloniki, Greece; aptzikas@yahoo.com; 7 Second Department of Cardiology, Faculty of Health Sciences, School of Medicine, General Hospital ‘Hippokration’, Aristotle University of Thessaloniki, 54642 Thessaloniki, Greece; fragakis.nikos@gmail.com (N.F.); aantoniadis@gmail.com (A.P.A.); 8 Structural and Valvular Center of Excellence, Marcus Heart Valve Center, Piedmont Heart Institute, Atlanta, GA 30309, USA; mvannan2560@gmail.com; 9 University of Rennes, CHU Rennes, Inserm, LTSI—UMR 1099, F-35000 Rennes, France; erwan.donal@gmail.com

**Keywords:** transesophageal echocardiography, mini-TEE probe, three-dimensional echocardiography, transcatheter interventions

## Abstract

**Background:** Transesophageal echocardiography (TEE) is a vital diagnostic tool in clinical practice, particularly in transcatheter interventions where it aids in both pre-operative planning and intra-operative guidance. Traditional TEE probes often require general anesthesia due to patient discomfort. However, the development of miniaturized TEE probes presents a promising alternative, enabling routine examinations and interventions with minimal sedation. This study evaluates the feasibility of performing a complete 2D/4D TEE protocol with the new 4D mini-TEE probe in the echocardiography department and its application in transcatheter interventions. **Methods:** This is a retrospective study that included 30 consecutive patients from two high-volume European hospitals (Interbalkan Medical Center, Thessaloniki, Greece, and Rennes University, France) that underwent TEE or transcatheter interventions. The new 4D mini-TEE 9VT-D probe (GE Healthcare) was utilized. The quality of the images and the tolerance of the probe were assessed in the cath lab during interventions and in the echocardiography department during routine TEE examinations. **Results:** Direct comparison of the 4D mini-TEE probe with the standard 6VT-D probe confirmed the excellent image quality of this new pediatric probe. Most of the patients required minimal sedation or local oropharyngeal anesthesia, with satisfactory tolerance reported. Most of the transcatheter procedures did not require general anesthesia and intubation, resulting in shorter procedural time. Both 2D and 4D imaging modalities offered adequate intra-operative guidance for transcatheter procedures. **Conclusions:** The 4D mini-TEE probe delivers exceptional imaging capabilities for routine examinations and transcatheter interventions without needing sedation. Its use reduces esophageal trauma and the need for general anesthesia, enhancing patient comfort and safety.

## 1. Introduction

Transesophageal echocardiography (TEE) is an invaluable diagnostic tool for structural heart diseases, offering high-quality images suitable for routine echocardiographic examinations [1,2,3]. Its significance extends to transcatheter interventions, where it plays a pivotal role from pre-operative planning to intra-operative guidance and final result assessment [4,5,6,7]. Effective collaboration between the imager and interventionist is crucial, with real-time demonstration of procedural steps facilitated by two- and three-dimensional (3D) echocardiographic images [8,9]. Notably, 3D echocardiography enables precise device sizing, exhibiting a strong correlation with other imaging modalities such as cardiac computed tomography (CT) [10].

Despite the generally favorable safety profile of adult TEE probes, they may cause minimal complications, including oropharyngeal trauma, esophageal lesions, and post-examination symptoms such as throat hoarseness or chest pain [11,12,13]. These complications are related to the thickness of the adult TEE probe, the manipulations of the probe during the examination, and probe-generated heat. The standard adult TEE probes are often poorly tolerated in non-sedated patients, limiting their prolonged use. While routine TEE examinations typically last 10–15 min and do not necessitate deep sedation, transcatheter interventions last longer, necessitating general anesthesia (GA) as standard practice in order to mitigate patient discomfort. However, GA requires the presence of an anesthesiologist and results in extended procedural and hospitalization times, along with an increased risk of anesthesia-related complications.

Intra-cardiac echocardiography (ICE) is as a potential solution to the limitations of adult TEE probes [14]. However, ICE is an invasive tool requiring additional venous access and has additional substantial costs. Another alternative, the micro-TEE probe (10T-D GE Healthcare), lacks support for three-dimensional imaging, which is imperative for most transcatheter procedures [15,16,17].

The objective of this study is to assess the feasibility of employing the new 4D mini-TEE probe for both routine examinations and guidance of transcatheter procedures, while evaluating the quality of the obtained images.

## 2. Materials and Methods

This is a multi-center retrospective analysis that assessed the feasibility of (1) using the 4D mini-TEE probe in everyday practice and (2) guiding transcatheter interventions. Our cohort consisted of 30 consecutive patients who either visited the echocardiography department or were scheduled for a transcatheter procedure. Interbalkan Medical Center and the University Hospital of Rennes participated in the study and the registry was approved by their local ethics and scientific committees. All TEE examinations were conducted by experienced imaging specialists with the new 9VT-D, 4D mini-TEE probe that has recently been made commercially available. All examinations were performed with the Vivid E95 machine and were stored and post-processed in EchoPAC workstations. Standard 2D/4D examination protocols [3,7] were acquired for complete assessment of the patients, for pre-operative planning and intra-operative guidance. For qualitative assessment of the images, a 5-point score was used (1: non-interpretable images, 2: poor quality but interpretable, 3: average quality, 4: good quality, 5: excellent quality) that was answered immediately after the end of the TEE and was internally reviewed by three experienced echocardiographers (K.P., I.I., E.D.).

### 2.1. Comparison Between Mini-TEE and Standard Adult TEE Probe

To assess the image quality of the mini-TEE probe and ensure the accuracy of findings, a limited number of patients were selected for examination using both the adult 6VT-D and pediatric mini-TEE 9VT-D probes. Prior to the examination, patients were duly informed about the procedure and provided their consent. The protocol dictated the initial examination with the adult probe followed by the insertion and utilization of the pediatric probe to capture identical images. To increase patient comfort during probe insertion, mild sedation with midazolam and local application of lidocaine to the oropharynx were administered.

### 2.2. Echocardiography Department Transesophageal Echocardiography

For routine TEE examinations, the anesthesia protocol comprised the local application of lidocaine at the posterior oropharynx, coupled, for some, with minimal sedation using 1–2 mg of midazolam to ensure patients remained calm yet awake throughout the procedure. In instances where patients exhibited intolerance to the probe, higher doses of midazolam (5–10 mg) were administered based on individual response levels.

Probe insertion into the esophagus involved the utilization of an endocavity probe-cover coated with lubricant gel at the tip, aiming at preventing artifacts from partial attachment of the mini probe to the esophageal wall. To assess probe tolerance, a 5-point scoring system was employed, with patients completing a questionnaire following the examination. Scores ranged from 1 (indicating probe intolerance necessitating termination of the examination) to 5 (signifying no discomfort experienced during or after the TEE procedure) (1: non-tolerable probe/termination of examination, 2: major discomfort during TEE, 3: major discomfort immediately after TEE with chest pain/bleeding, 4: minor discomfort, 5: no discomfort during or post-TEE).

### 2.3. Intra-Procedural Transesophageal Echocardiography

For transcatheter procedures, the preferred approach involved sedation without the need for general anesthesia or intubation. This decision was contingent upon factors such as the duration of the procedure, patient tolerance of the probe, and their ability to remain still during the operation, thereby assisting the interventionist in completing the procedure effectively. During the insertion of the probe into the esophagus for transcatheter procedures, an endocavity probe-cover coated with lubricant gel was once again utilized. Unlike routine TEE examinations where patients typically lie in the decubitus position, for transcatheter procedures, all patients were positioned supinely to facilitate probe insertion. Probe tolerance was assessed at the conclusion of the procedure through a straightforward inquiry regarding the patient’s level of cooperation or necessity for intubation.

## 3. Results

### 3.1. Comparison Between Mini-TEE and Standard Adult TEE Probe

The quality of images obtained with the 9VT-D pediatric probe and the accuracy of findings were assessed through examination of the first three patients. Each patient underwent a comprehensive TEE protocol using both the pediatric 9VT-D and adult 6VT-D probes, allowing for direct comparison of findings and image quality. Throughout the procedure, patients received local oropharyngeal lidocaine and mild sedation with midazolam to ensure tolerance.

**Figure 1:**
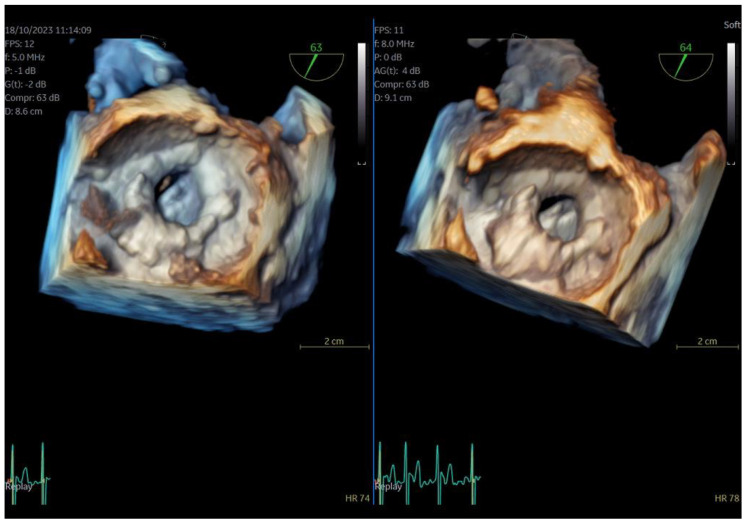
Direct comparison of 3D volume −rendered “en face” images of a metallic prosthetic mitral valve with occluded disk. Left image corresponds to 6VT−D adult probe and right image corresponds to 9VT−D pediatric probe.

**Figure 2:**
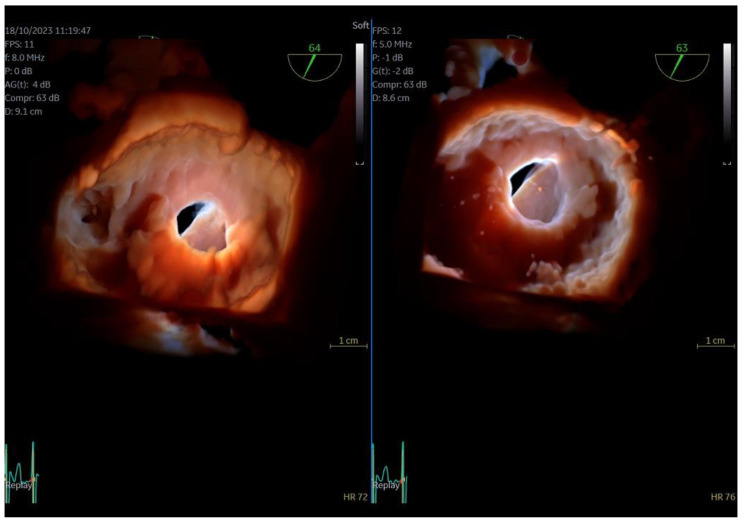
Direct comparison of 3D volume-rendered “en face” images of a metallic prosthetic mitral valve with occluded disk. Left image corresponds to 6VT−D adult probe and right image corresponds to 9VT−D pediatric probe. Images enhanced with “photorealistic method” with Flexilight application (GE Healthcare).

The adult probe was initially inserted for complete 2D/4D image acquisition, followed by the insertion of the pediatric probe while the patient remained under sedation. Remarkably, insertion of the pediatric probe was effortless in all cases, requiring no additional sedation. Despite encountering challenging diagnostic scenarios in all three cases (1st patient: combined severe aortic stenosis and severe mitral regurgitation (MR) due to P1 scallop prolapse, 2nd patient: malfunction of metallic mitral valve prosthesis with an occluded disk, 3rd patient: bioprosthetic aortic valve with paravalvular leak and significant MR in previously MV repair with a complete ring), the pediatric probe consistently provided high-quality images comparable to those obtained with the standard adult probe (Figure 1 and Figure 2, video S4). Notably, there was no compromise in 2D and 4D spatial and temporal resolution, with only minor differences observed compared to the adult 6VT-D probe (Table 1). Evaluation using the 5-point scoring system consistently yielded an average score close to 5 for all three cases, indicating excellent image quality (Table 1).

### 3.2. Echocardiography Department Transesophageal Echocardiography

Following the initial comparison between the pediatric and adult probes, where confidence in the accuracy of the 9VT-D probe was established, the rest of the consecutive patients underwent the TEE protocol solely with the pediatric probe. Most patients received local anesthesia of the posterior pharynx with lidocaine but only four (4) of them required minimal doses of midazolam (1–2 mg) due to slight discomfort in order to ensure they remained cooperative throughout the procedure. With the exception of one patient who experienced intense pharyngeal reflex and required sedation with 5 mg of midazolam, all other patients tolerated the mini-TEE probe well (Table 2).

Our initial experience further showed that in a small number of patients and especially the younger ones with pharyngeal reflex, left lateral decubitus position was suboptimal for the introduction of the probe. The introduction in these patients was much easier in the sitting position and they were asked to swallow the probe by themselves with a very limited involvement of the physician or the nurse. Local anesthesia could also be unnecessary for a significant number of patients in the future after we have obtained experience of introducing this new mini probe.

**Figure 3:**
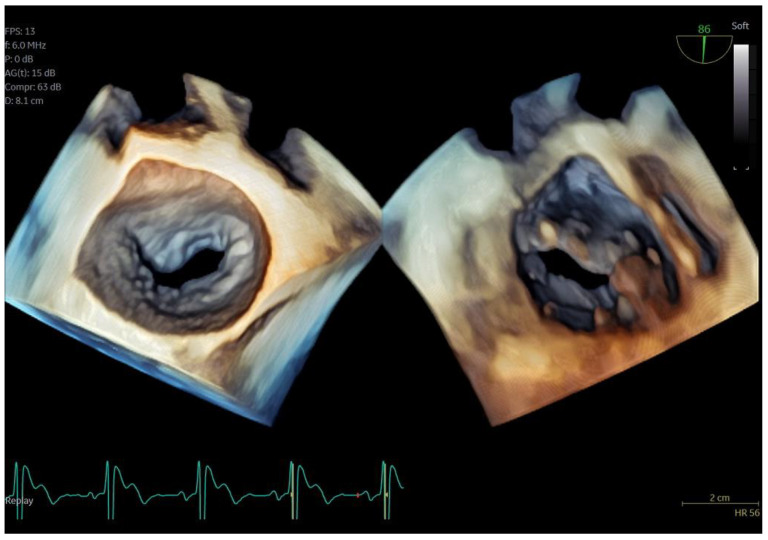
Dual crop 3D volume-rendered atrial (**left**) and ventricular (**right**) views of normal mitral valve.

**Figure 4:**
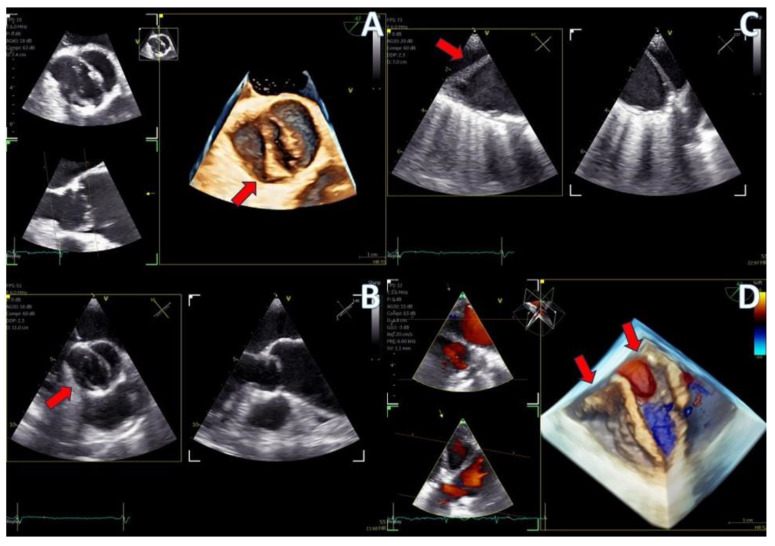
(**A**) A 4D demonstration of a true−bicuspid aortic valve (red arrow), (**B**) Biplane 2D demonstration of a true−bicuspid aortic valve (red arrow), (**C**) Chronic dissection of descending aorta (red arrow showing the wall of the true lumen), (**D**) 3D volume-rendered color Doppler image showing the true (right arrow) and the false lumen (left arrow).

Regarding imaging quality, almost all patients exhibited excellent images without any compromise in resolution, obviating the need to confirm findings using the standard TEE probe (Figure 3 and Figure 4, video S5). Despite the one patient requiring additional sedation due to intense pharyngeal reflex, the quality of images obtained remained consistent across all cases. In 22 cases out of 30 included in this study, the 2D and 3D imaging quality received a score of 5 suggesting excellent imaging totally equal to the one acquired with the adult probe. In four cases, 2D and 3D images were commented as good with a score of 4 and in another four cases, while the 2D images were excellent, 3D images were again mentioned to be good, almost equal to the standard probe but not exceptional enough to receive a grade of 5 (Table 3).

### 3.3. Intra-Procedural Transesophageal Echocardiography

The new 4D mini-TEE probe was utilized in eight consecutive patients undergoing transcatheter procedures at our clinic. Among them, four patients underwent cryo-ablation for atrial fibrillation, two underwent patent foramen ovale (PFO) closure, one underwent atrial septal defect (ASD ostium secundum) closure, and one underwent left atrial appendage (LAA) closure.

Despite the protocol recommending minimal sedation and avoidance of intubation, two of the “ablation” patients were unable to tolerate the probe, necessitating intubation and administration of general anesthesia. Additionally, the patient scheduled for LAA closure required general anesthesia due to the detection of thrombus formation at the distal part of the appendage at the onset of the procedure, posing safety concerns for the interventionist. The decision to proceed under general anesthesia was made to minimize risk and ensure patient safety. Conversely, the remaining “ablation” patients, as well as those undergoing ASD and PFO closure, tolerated the mini-TEE probe well and completed the intervention with mild sedation only. The average duration of probe insertion into the esophagus for all procedures was 29 min (range 20–32 min). None of the patients reported post-operative discomfort, and no esophageal trauma was observed. The anesthesiologist noted that while it was easier to awaken patients who did not receive general anesthesia, maintaining sufficient sedation for the duration of the procedure was more demanding. Despite the challenges encountered, there were no anesthesia-related complications, and all patients adhered to the standard 24 h post-procedural hospitalization protocol. However, same-day discharge can be considered for patients who do not require intubation, offering an attractive and cost-effective option.

**Figure 5:**
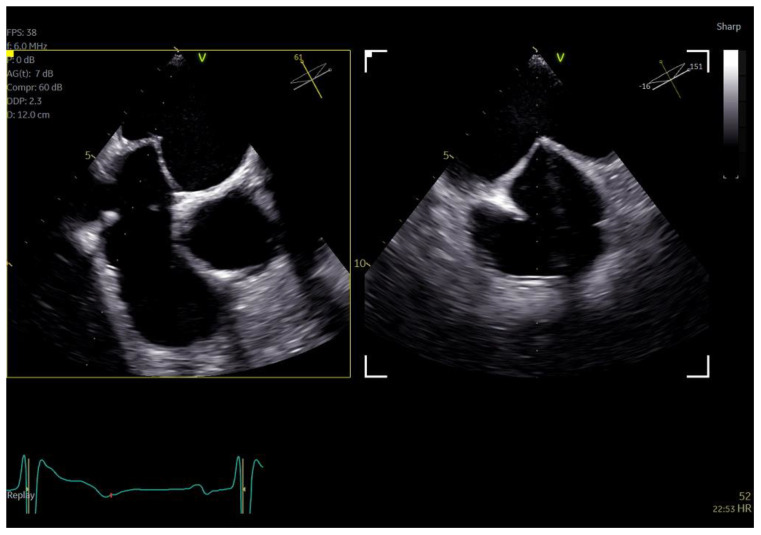
Biplane images of tending of the atrial septum during trans−septal puncture for atrial fibrillation cryo−ablation.

**Figure 6:**
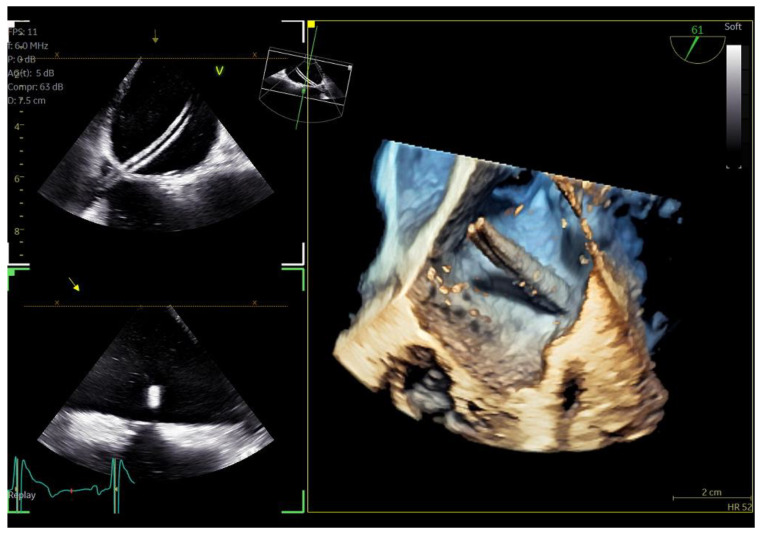
Azimuth level (**top left**), elevation level (**bottom left**), and 3D volume-rendered image (**right**) of an SL0 catheter for the guidance of cryo-ablation procedure.

**Figure 7:**
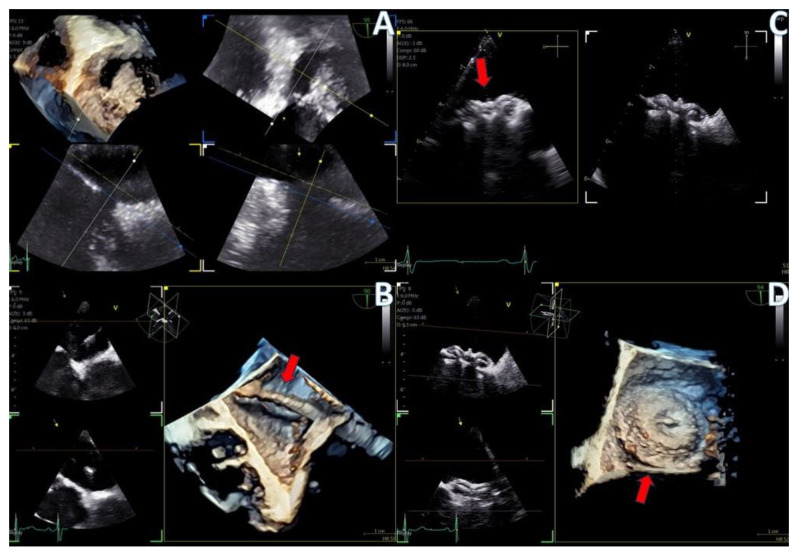
Secundum ASD closure case; (**A**) Flexislice method for measurements with both 2D and 4D images, (**B**) guide catheter through the defect (red arrow), (**C**) biplane 2D images showing the implanted ASD occluder, (**D**) 3D volume-−rendered image showing the final result with an ASD occluder.

**Figure 8:**
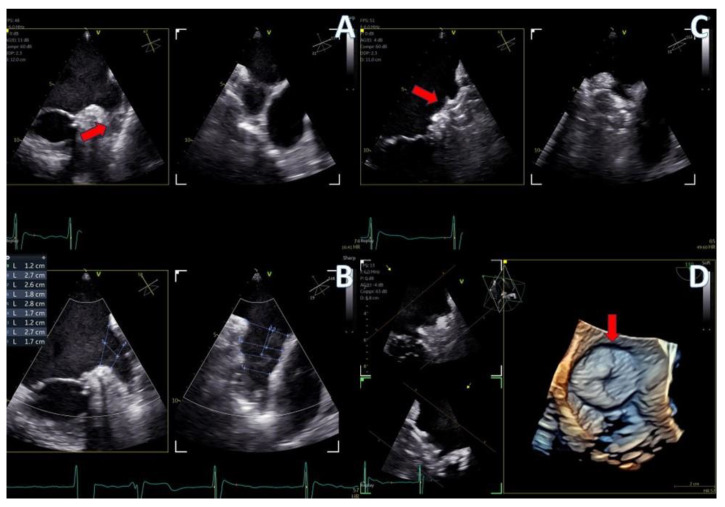
LAA closure case; (**A**) biplane images of the LAA showing the presence of distal thrombus (red arrow), (**B**) measurements of the ostium and the landing zone with biplane imaging, (**C**) Biplane images of the implanted AMULET device (red arrow), (**D**) 3D volume−rendered image showing a view of the implanted AMULET device (red arrow).

**Figure 9:**
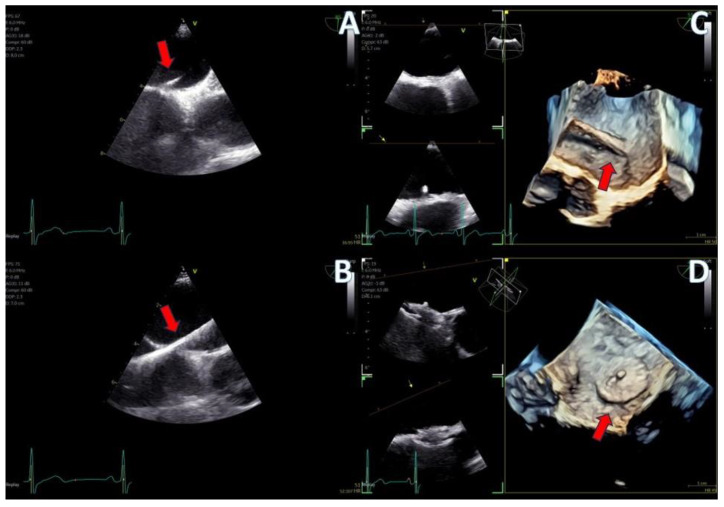
PFO closure case; (**A**) J wire through the PFO tunnel (red arrow), (**B**) stiff wire for guidance through the PFO tunnel (red arrow), (**C**) 3D volume−rendered view of the guide catheter through the PFO tunnel (red arrow), (**D**) final result with left atrial view of the PFO Amplatzer occluder (red arrow).

The imaging capabilities of the 4D mini-TEE probe were consistently excellent across all procedures (Figure 5, Figure 6, Figure 7, Figure 8 and Figure 9). Trans-septal puncture in ablation cases was facilitated by biplane imaging (Figure 5), demonstrating a nice tending of the septum. For the guidance of wires to the pulmonary veins, both 2D and 4D volume-rendered images were utilized (Figure 5 and Figure 6), expediting the procedure. The TEE probe was removed after the insertion of the cryo-ablation catheter into the LA.

In the case of LAA closure (Figure 8, video S3), 3D tools (MPR-flexislice) aided in accurate sizing of the ostium and the landing zone (important for AMULET device), particularly in the presence of distal thrombus where contrast infusion was contraindicated. The final result was satisfactory, with no leakage observed and a stable device placement was confirmed with a tug test.

PFO closure cases (Figure 9, video S2) benefited from the probe’s excellent imaging, enabling precise wire insertion and providing anatomical criteria for device sizing. A bubble contrast study was performed at the beginning and at the end of the procedure for any remaining shunt. The stability of the device was confirmed with a wiggle test. Similarly, in the ASD case (Figure 7, video S1), the probe facilitated visualization of all defect rims and accurate sizing of the oval-shaped defect (with MPR) confirmed with balloon sizing and stop-flow technique and ensured a successful outcome with no residual shunt observed.

Overall, the 4D mini-TEE probe demonstrated exceptional imaging capabilities and played a pivotal role in guiding transcatheter interventions with precision and efficacy.

## 4. Discussion

Our study represents a pioneering effort in evaluating the new 4D mini-TEE probe (9VT-D GE Healthcare) across diverse adult patient populations, encompassing both routine TEE examinations and transcatheter procedures. Dr. Sanchis et al. have recently published their experience with this particular probe in a small number of LAA occlusion cases with excellent feedback in terms of patient’s tolerance and imaging quality [18]. Dr Karcenty et al. have also published data from the use of this particular probe in a pediatric population, showing superior quality of images compared with other 2D pediatric probes [19]. While previous studies have included procedures using miniaturized TEE probes [15,16,17,20,21], ours is unique by the fact that this is the first commercially available probe boasting full 3D options and capable of supporting all TEE examinations and transcatheter procedures in adults. The added value of 3D echocardiography lies in its capacity for precise device sizing and improved comprehension of cardiac anatomy. Notably, biplane imaging facilitates trans-septal puncture, thereby reducing procedural time. Multi-planar reconstruction (Flexislice method) serves as a vital tool for sizing in cases such as secundum ASDs and LAAs, where precise structural dimensions are imperative [4].

Our study demonstrates that the new 4D mini-TEE 9VT-D probe effectively addresses the limitations of the adult TEE probe. The introduction of the probe requires less effort, especially in non-sedated patients that are asked to swallow it, under the physician’s supervision. The role of the physician continues with the manipulation of the probe with the patient lying down in a left decubitus position. Direct comparison of images between the pediatric and adult probes confirmed the exceptional imaging quality achieved with the mini probe. The inclusion of 3D tools facilitates both routine examinations and transcatheter interventions, rendering this probe superior to previous mini-TEE/micro-TEE 2D probes and intra-cardiac echocardiography (ICE). While ICE may emerge as an attractive alternative option in the future, its current limitations in terms of cost and image resolution preclude it from matching the 3D imaging capabilities of the 9VT-D probe [14].

Moreover, the thin and flexible nature of the 9VT-D probe ensures good tolerability during the examination even in non-sedated patients. Our study analyzed thirty patients but only nineteen were included in the scoring system for the tolerance of the probe since the first three patients received sedation in order to complete the protocol with comparison of both probes, and another eight patients underwent transcatheter interventions with the presence of an anesthesiologist. Of these nineteen patients that completed the TEE examination without sedation, only one mentioned major discomfort and did not tolerate the probe, while four others reported minor discomfort. The remaining 14 patients tolerated this mini probe well and graded their experience with a score of 5.

This new probe is probably a tool that will become necessary for large tertiary centers where frail patients are often referred for diagnosis that could be suitable for percutaneous treatment. The greatest benefit is observed in transcatheter procedures, where the full array of 3D options can be leveraged without requiring intubation and general anesthesia. This results in reduced procedural time, avoidance of anesthesia-related complications, and the potential for same-day discharge, particularly advantageous for high-surgical-risk patients undergoing transcatheter procedures. Notably, our study revealed no complications from the esophagus or the administered anesthesia.

In summary, our study underscores the remarkable clinical utility and patient benefits afforded by the 4D mini-TEE 9VT-D probe, positioning it as a valuable tool in contemporary echocardiography practice, particularly in the era of transcatheter interventions.

### Limitations

While our experience with the mini-TEE probe in routine practice has generally been favorable, there are certain issues that warrant mention. Unlike the standard adult probe, which is thick and rigid, facilitating its advancement past the tongue and posterior pharynx, the mini-TEE probe is thin and flexible. This can result in instances where the probe curls into the oral cavity, resembling a naso-gastric Levin tube. In one intubated patient, the anesthesiologist had to utilize a laryngoscope to aid in probe insertion into the esophagus. Additionally, in one awake patient who exhibited poor tolerance to the probe, standard doses of sedation were necessary to complete the examination.

Another limitation is the current cost of the mini-TEE probe which is 1.5 times higher than that of the standard adult 4D probe (6VT-D). Our pilot experience provides convincing evidence in favor of the use of the mini-TEE despite its higher cost. Future studies will have to further investigate the clinical advantages and the long-term benefits of this adult probe by targeting a larger population. A randomized control trial using the standard adult probe could further assess the tolerance of the new probe by using the same scoring scale. Finally, this new probe could be further evaluated in a direct comparison with other pediatric 2D-TEE probes that are already established in standard practice.

## 5. Conclusions

The introduction of the new 4D mini-TEE probe represents a significant advancement in echocardiography, offering excellent imaging capabilities and encompassing all the essential 2D/4D options required for a comprehensive TEE protocol and guidance of transcatheter interventions. Notably, the utilization of this probe might lead to a reduction in esophageal and anesthesia-related complications. However, further validations are needed.

## Figures and Tables

**Table 1:** Direct comparison of imaging quality in patients examined with both 6VT-D and 9VT-D probe. Scoring system: 1: non-interpretable images, 2: poor quality but interpretable, 3: average quality, 4: good quality, 5: excellent quality. 2D, two-dimensional; PWD, pulsed-wave Doppler; CWD, continuous-wave Doppler; 3D, three-dimensional.

Probe Used	6VT-D						9VT-D					
Method for Scoring	2D	2D Color	PWD	CWD	3D	Average Score	2D	2D Color	PWD	CWD	3D	Average Score
1st patient	5	5	5	5	5	5	5	5	5	5	4	4.8
2nd patient	5	5	5	5	5	5	5	5	5	5	5	5
3rd patient	5	5	5	5	5	5	4	5	5	5	4	4.6

**Table 2:** Tolerance of probe during examinations in the echocardiography department. Five-point scoring system. 1: non-tolerable probe/termination of examination, 2: major discomfort during TEE, 3: major discomfort immediately after TEE with chest pain/bleeding, 4: minor discomfort, 5: no discomfort during or post-TEE.

Score	Probe Tolerance (Number of Patients)
	Total Number of Patients = 19
1	0
2	1
3	0
4	4
5	14

**Table 3:** Scoring of imaging quality of the new 4D mini-TEE probe according to different echocardiographic modalities: 1: non-interpretable images, 2: poor quality but interpretable, 3: average quality, 4: good quality, 5: excellent quality. 2D, two-dimensional; PWD, pulsed-wave Doppler; CWD, continuous-wave Doppler; 3D, three-dimensional.

Echocardiographic Modality	Number of Patients	1 Non- Interpretable	2 Poor	3 Average	4 Good	5 Excellent	Average Score
2D	30	0	0	0	4	26	4.87
2D color	30	0	0	0	0	30	5
PWD	30	0	0	0	0	30	5
CWD	30	0	0	0	0	30	5
3D	30	0	0	0	8	22	4.73
3D color	30	0	0	0	8	22	4.73

## Data Availability

All data supporting this article are available upon reasonable request.

## Supplementary Materials

The following supporting information can be downloaded at: https://www.mdpi.com/article/10.3390/jcm13216450/s1, Video S1: Atrial septal defect closure case, Video S2: Patent foramen ovale closure case, Video S3: Left atrial appendage closure case, Video S4: Metallic mitral valve assessment, Video S5: Bicuspid aortic valve with chronic descending aortic dissection.

## References

[1] Hahn R.T., Abraham T., Adams M.S., Bruce C.J., Glas K.E., Lang R.M., Reeves S.T., Shanewise J.S., Siu S.C., Stewart W. (2013). Guidelines for performing a comprehensive transesophageal echocardiographic examination: Recommendations from the American Society of Echocardiography and the Society of Cardiovascular Anesthesiologists. J. Am. Soc. Echocardiogr..

[2] Nicoara A., Skubas N., Ad N., Finley A., Hahn R.T., Mahmood F., Mankad S., Nyman C.B., Pagani F., Porter T.R. (2020). Guidelines for the Use of Transesophageal Echocardiography to Assist with Surgical Decision-Making in the Operating Room: A Surgery-Based Approach: From the American Society of Echocardiography in Collaboration with the Society of Cardiovascular Anesthesiologists and the Society of Thoracic Surgeons. J. Am. Soc. Echocardiogr..

[3] Papadopoulos C.H., Kadoglou N.P., Theodosis-Georgilas A., Papadopoulos K.G., Rallidis L., Loizos S., Karabinos I., Kassinos N., Sahpekidis V., Chrysoheris M. (2024). Practical guidance and clinical applications of transoesophageal echocardiography. A position paper of the working group of echocardiography of the Hellenic Society of Cardiology. Curr. Probl. Cardiol..

[4] Faletra F.F., Saric M., Saw J., Lempereur M., Hanke T., Vannan M.A. (2021). Imaging for Patient’s Selection and Guidance of LAA and ASD Percutaneous and Surgical Closure. JACC Cardiovasc. Imaging.

[5] Silvestry F.E., Rodriguez L.L., Herrmann H.C., Rohatgi S., Weiss S.J., Stewart W.J., Homma S., Goyal N., Pulerwitz T., Zunamon A. (2007). Echocardiographic guidance and assessment of percutaneous repair for mitral regurgitation with the Evalve MitraClip: Lessons learned from EVEREST I. J. Am. Soc. Echocardiogr..

[6] Khalique O.K., Hahn R.T. (2017). Role of Echocardiography in Transcatheter Valvular Heart Disease Interventions. Curr. Cardiol. Rep..

[7] Papadopoulos C.H., Kadoglou N.P., Theodosis-Georgilas A., Karabinos I., Loizos S., Papadopoulos K.G., Chrysocheris M., Ninios V., Frogoudaki A., Drakopoulou M. (2024). Transoesophageal echocardiography beyond the echo-laboratory. An expert consensus paper of the working group of echocardiography of the hellenic society of cardiology. Hell. J. Cardiol..

[8] Bushari L.I., Reeder G.S., Eleid M.F., Chandrasekaran K., Eriquez-Sarano M., Rihal C.S., Maalouf J.F. (2019). Percutaneous Transcatheter Edge-to-Edge MitraClip Technique: A Practical “Step-by-Step” 3-Dimensional Transesophageal Echocardiography Guide. Mayo Clin. Proc..

[9] Lang R.M., Tsang W., Weinert L., Mor-Avi V., Chandra S. (2011). Valvular heart disease. The value of 3-dimensional echocardiography. J. Am. Coll. Cardiol..

[10] Coisne A., Pontana F., Aghezzaf S., Mouton S., Ridon H., Richardson M., Polge A.-S., Longère B., Silvestri V., Pagniez J. (2020). Utility of Three-Dimensional Transesophageal Echocardiography for Mitral Annular Sizing in Transcatheter Mitral Valve Replacement Procedures: A Cardiac Computed Tomographic Comparative Study. J. Am. Soc. Echocardiogr..

[11] O’shea J.P., Southern J.F., D’ambra M.N., Magro C., Guerrero J.L., Marshall J.E., Vlahakes G.V., Levine R.A., Weyman A.E. (1991). Effects of prolonged transesophageal echocardiographic imaging and probe manipulation on the esophagus—An echocardiographic-pathologic study. J. Am. Coll. Cardiol..

[12] Freitas-Ferraz A.B., Bernier M., Vaillancourt R., Ugalde P.A., Nicodème F., Paradis J.-M., Champagne J., O’hara G., Junquera L., del Val D. (2020). Safety of Transesophageal Echocardiography to Guide Structural Cardiac Interventions. J. Am. Coll. Cardiol..

[13] Ruf T., Heidrich F., Sveric K., Pfluecke C., Stephan A.-M., Strasser R., Wiedemann S. (2017). ELMSTREET (Esophageal Lesions during MitraClip uSing TRansEsophageal Echocardiography Trial). EuroIntervention.

[14] Alkhouli M., Hijazi Z.M., Holmes D.R., Rihal C.S., Wiegers S.E. (2018). Intracardiac Echocardiography in Structural Heart Disease Interventions. JACC Cardiovasc. Interv..

[15] Stec S., Zaborska B., Sikora-Frac M., Kryński T., Kułakowski P. (2011). First experience with microprobe transoesophageal echocardiography in non-sedated adults undergoing atrial fibrillation ablation: Feasibility study and comparison with intracardiac echocardiography. Europace.

[16] Brítez G.J., Sanchis L., Regueiro A., Sabate M., Sitges M., Freixa X. (2019). Minimally-invasive Transesophageal Echocardiography for Left Atrial Appendage Occlusion With a Latest-generation Microprobe. Initial Experience. Rev. Esp. Cardiol..

[17] Nijenhuis V.J., Alipour A., Wunderlich N.C., Rensing B.J.W.M., Gijsbers G., Berg J.M.T., Suttorp M.J., Boersma L.V.A., van der Heyden J.A.S., Swaans M.J. (2017). Feasibility of multiplane microtransoesophageal echocardiographic guidance in structural heart disease transcatheter interventions in adults. Neth. Heart J..

[18] Sanchis L., Regueiro A., Cepas-Guillén P., Sitges M., Freixa X. (2023). First experience of left atrial appendage occlusion using a 3D mini transoesophageal echocardiographic probe with conscious sedation. EuroIntervention.

[19] Karsenty C., Hadeed K., Pyra P., Guitarte A., Djeddai C., Vincent R., Dulac Y., Silagdze I., Gobin J., Combes N. (2023). Advancing paediatric cardiac imaging: A comprehensive analysis of the feasibility and accuracy of a novel 3D paediatric transoesophageal probe. Front. Cardiovasc. Med..

[20] Maarse M., Wintgens L.I., Klaver M.N., Rensing B.J., Swaans M.J., Boersma L.V. (2021). Transoesophageal echocardiography guidance with paediatric probes in adults undergoing left atrial appendage occlusion. EuroIntervention.

[21] Aminian A., Leduc N., Freixa X., Swaans M.J., Ben Yedder M., Maarse M., Sanchis L., Cepas-Guillen P., Cruz-González I., Blanco-Fernandez F. (2023). Left Atrial Appendage Occlusion Under Miniaturized Transesophageal Echocardiographic Guidance and Conscious Sedation: Multicenter European Experience. JACC Cardiovasc. Interv..

