# Supplementary material for: Initial Experience with the 4D Mini-TEE Probe in the Adult Population
Source: J Clin Med. 2024 Oct 28;13(21):6450. doi: 10.3390/jcm13216450 (PMC11546711; doi:10.3390/jcm13216450)

## Slide 1
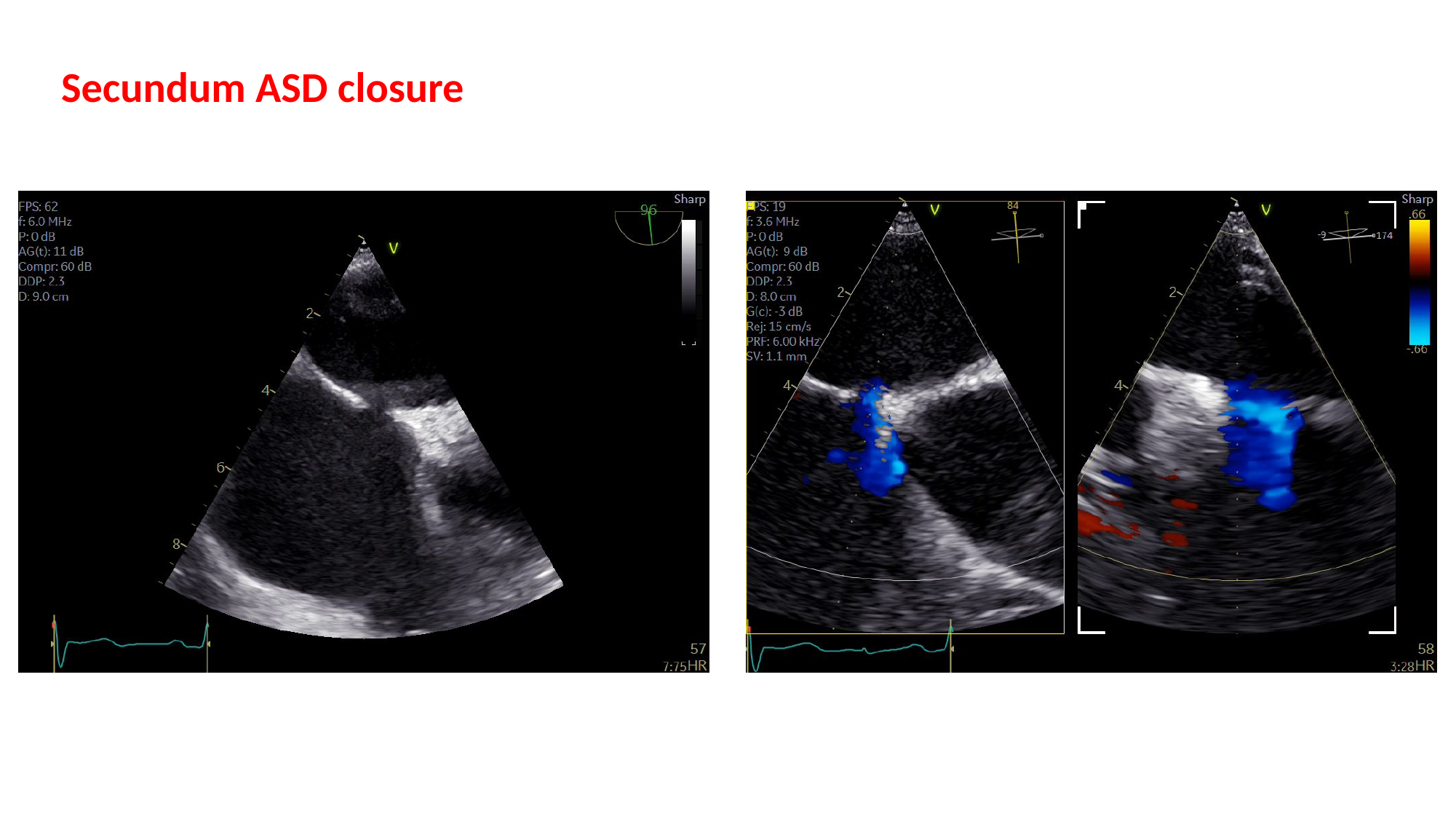

Secundum ASD closure

## Slide 2
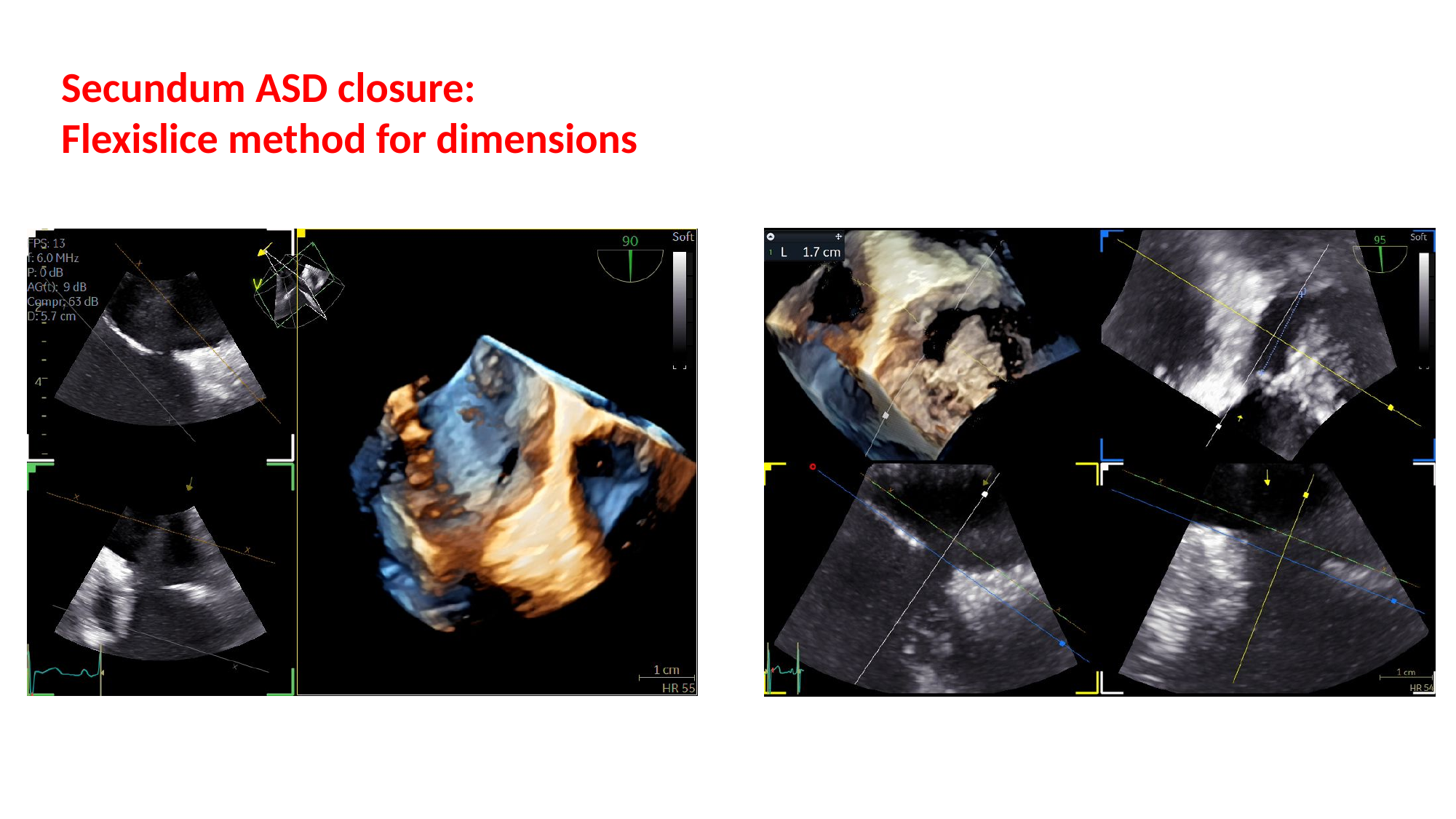

Secundum ASD closure:
Flexislice method for dimensions

## Slide 3
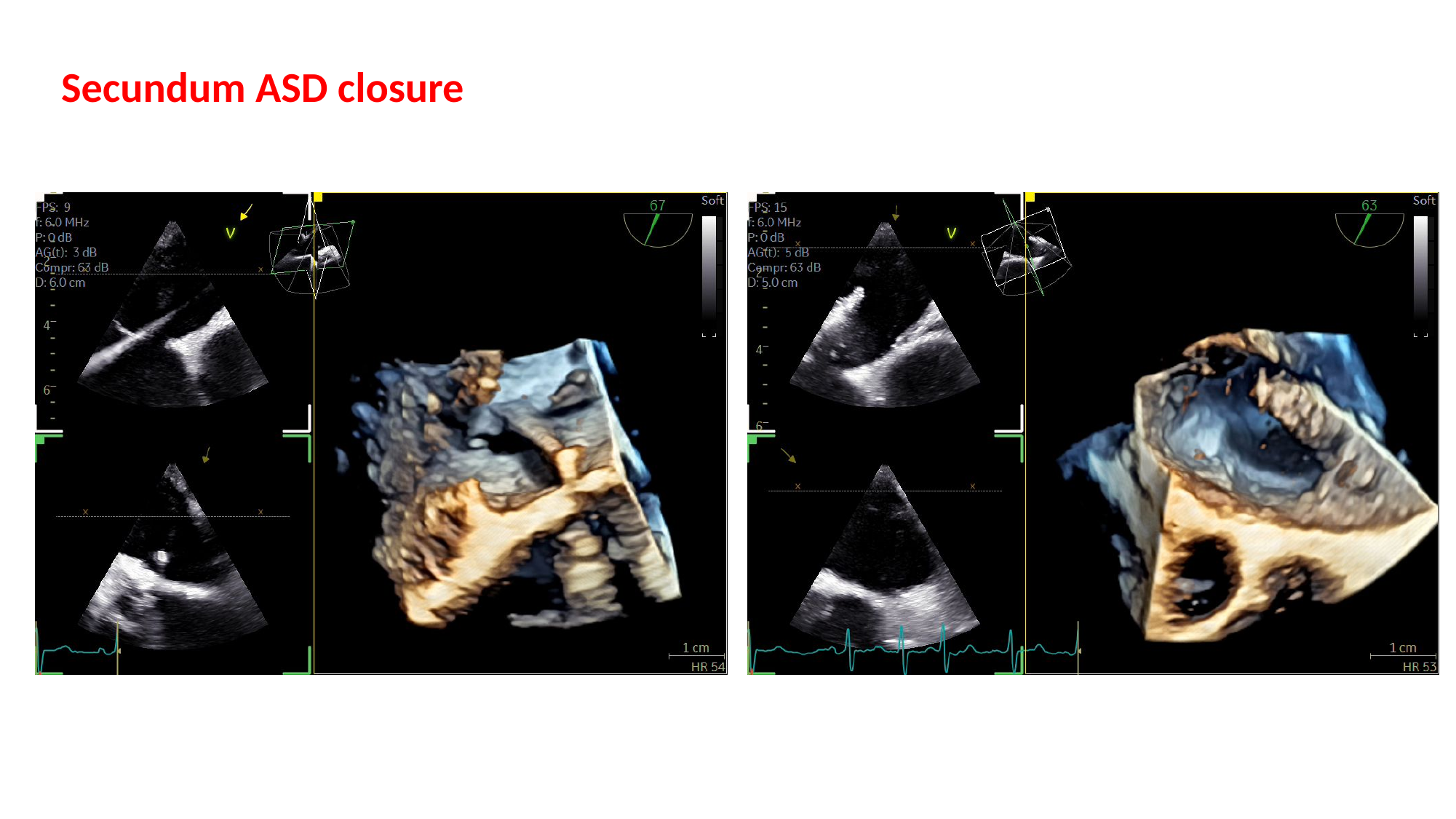

Secundum ASD closure

## Slide 4
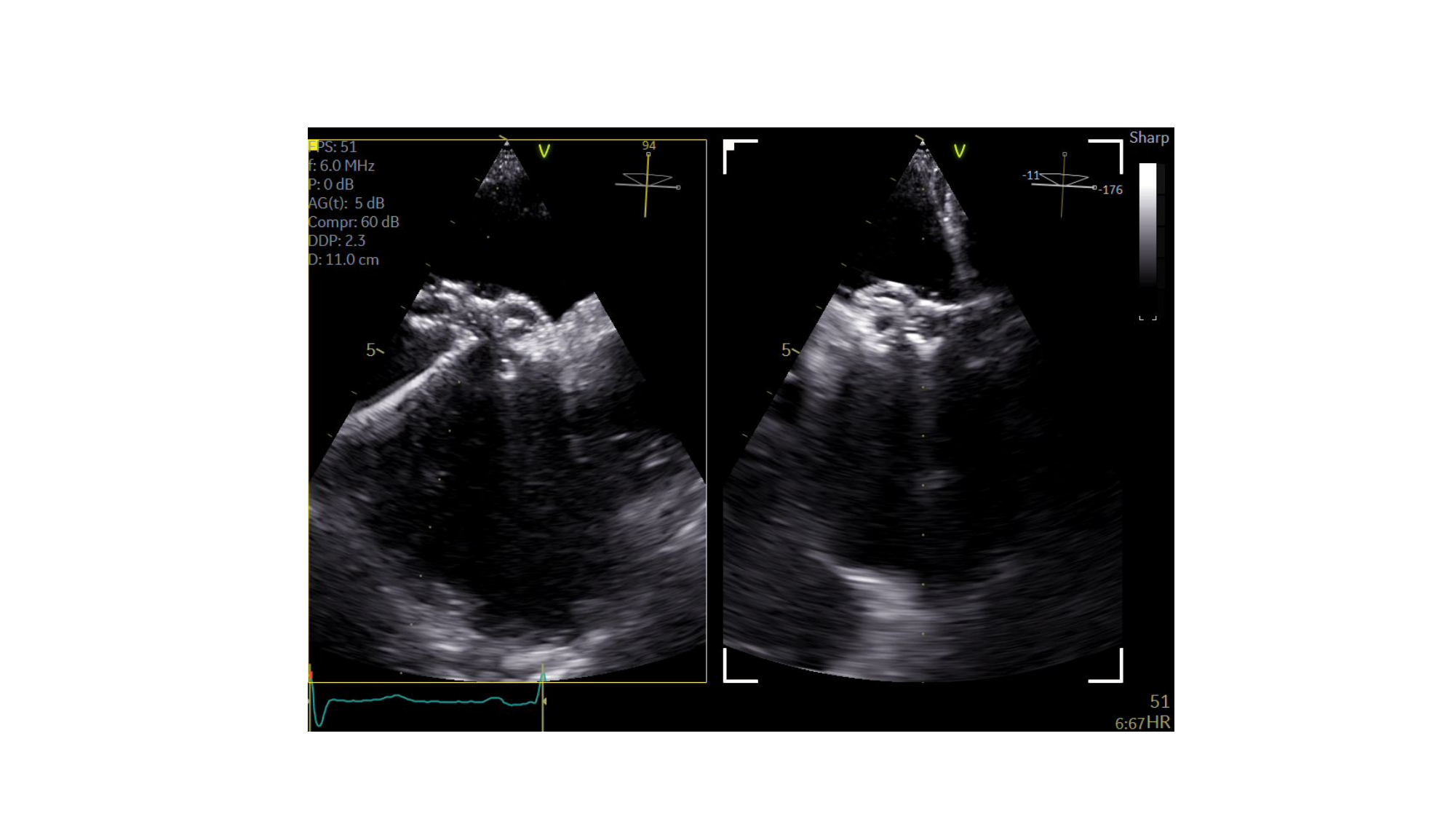

## Slide 5
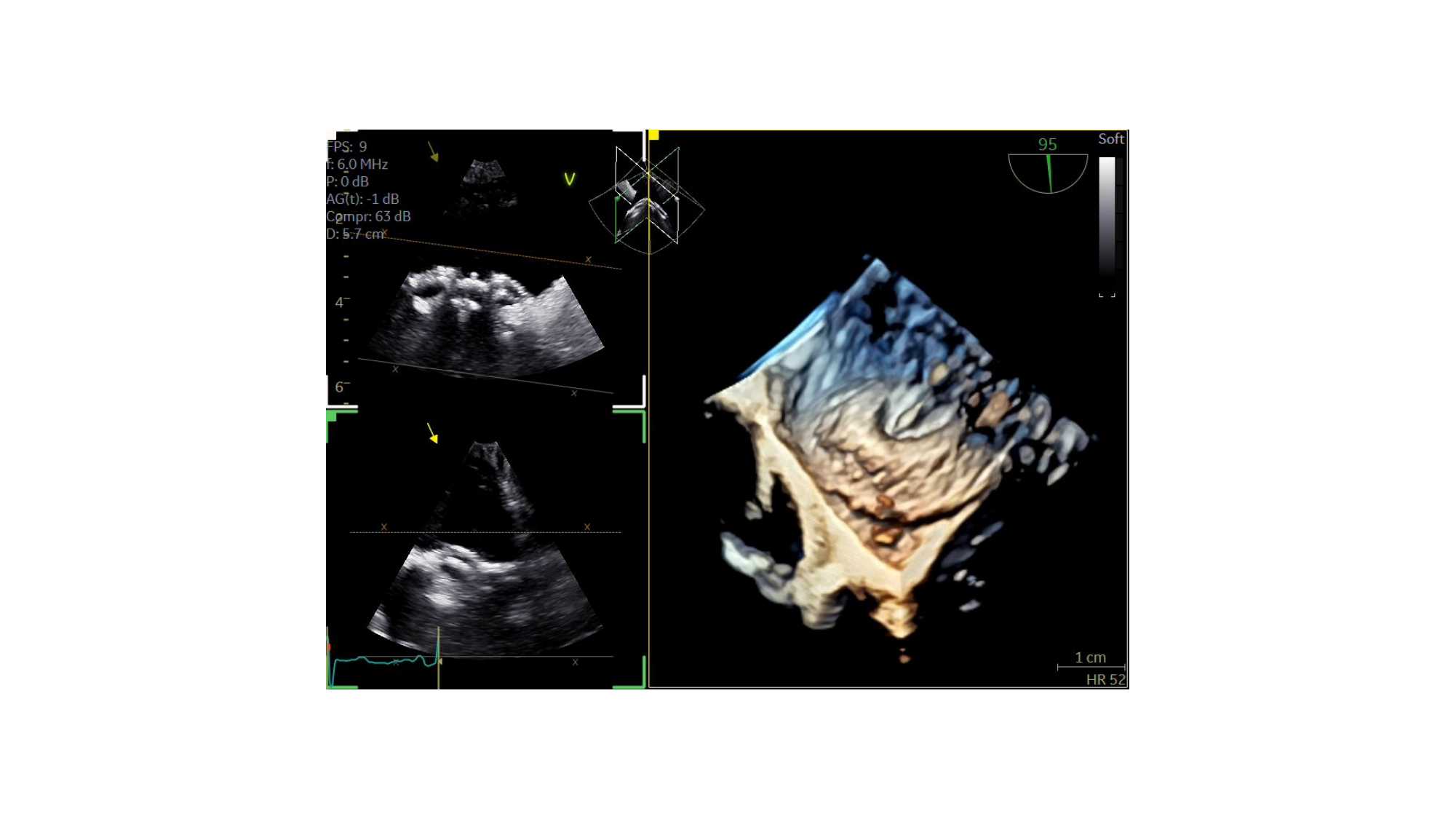

Supplement: Supplementary file 1 [file jcm-13-06450-s001.zip › VIDEO S1 ASD CLOSURE CASE.pptx]
