# Supplementary material for: Initial Experience with the 4D Mini-TEE Probe in the Adult Population
Source: J Clin Med. 2024 Oct 28;13(21):6450. doi: 10.3390/jcm13216450 (PMC11546711; doi:10.3390/jcm13216450)

## Slide 1
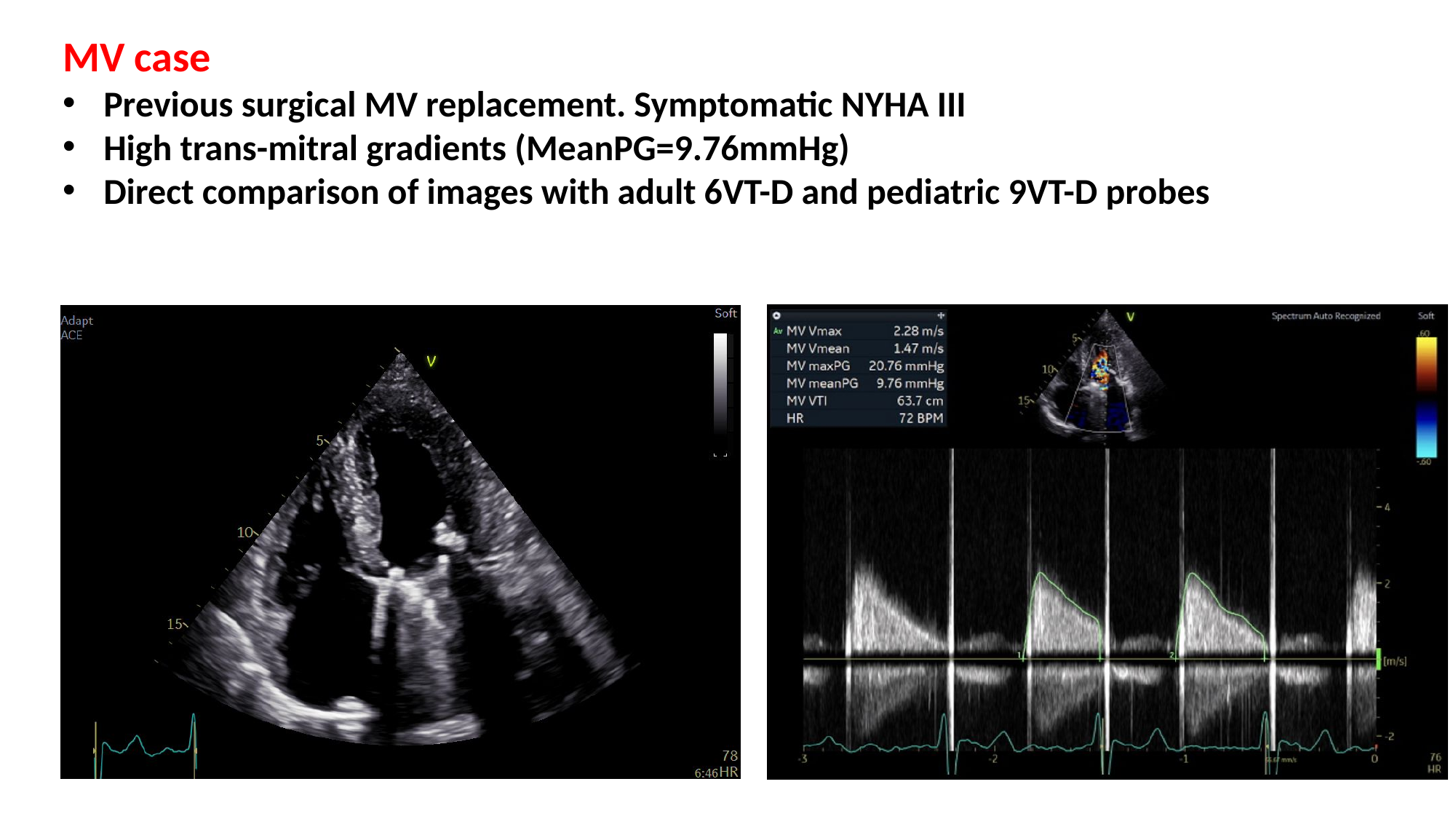

MV case
Previous surgical MV replacement. Symptomatic NYHA III
High trans-mitral gradients (MeanPG=9.76mmHg)
Direct comparison of images with adult 6VT-D and pediatric 9VT-D probes

## Slide 2
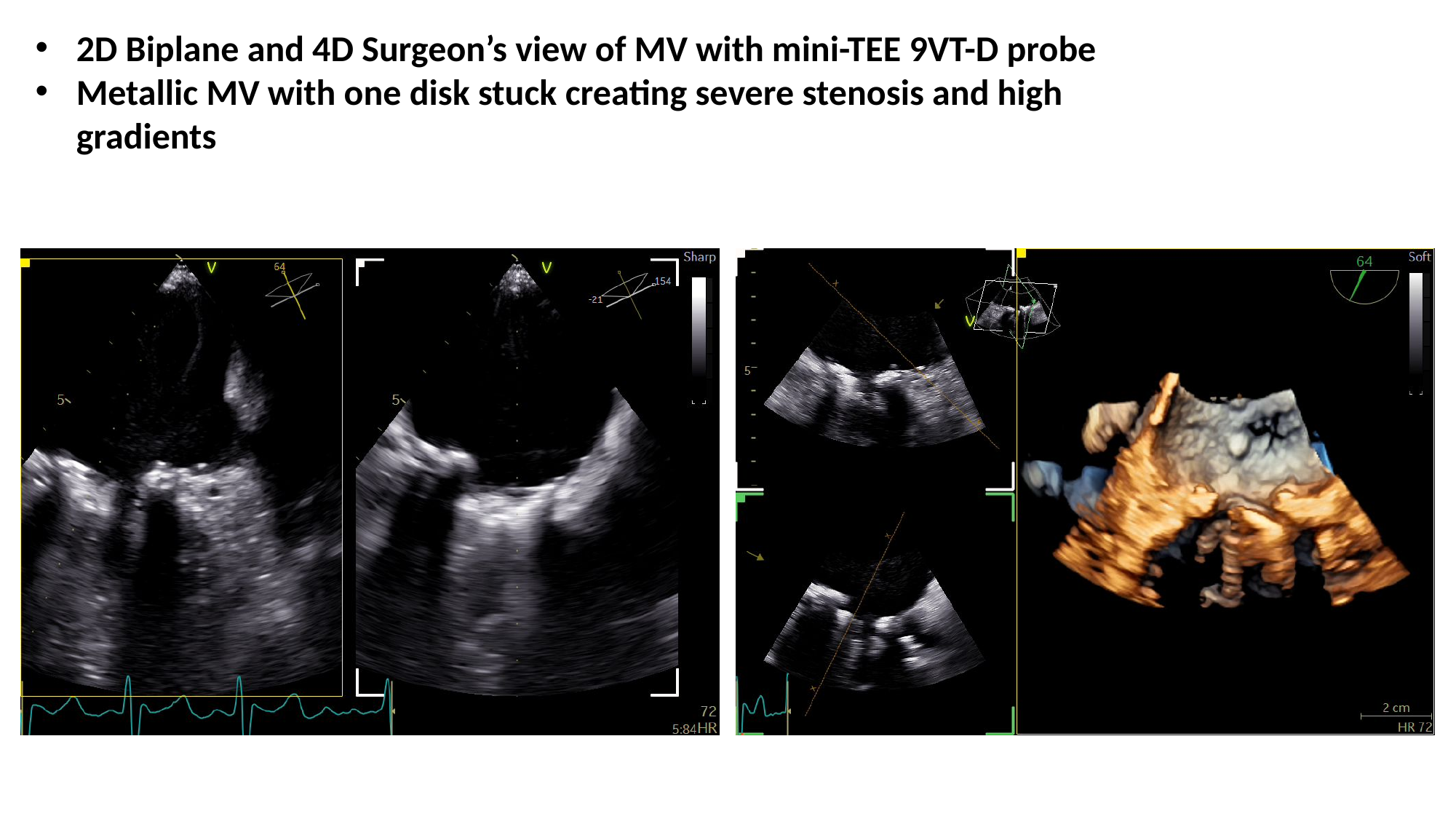

2D Biplane and 4D Surgeon’s view of MV with mini-TEE 9VT-D probe
Metallic MV with one disk stuck creating severe stenosis and high gradients

## Slide 3
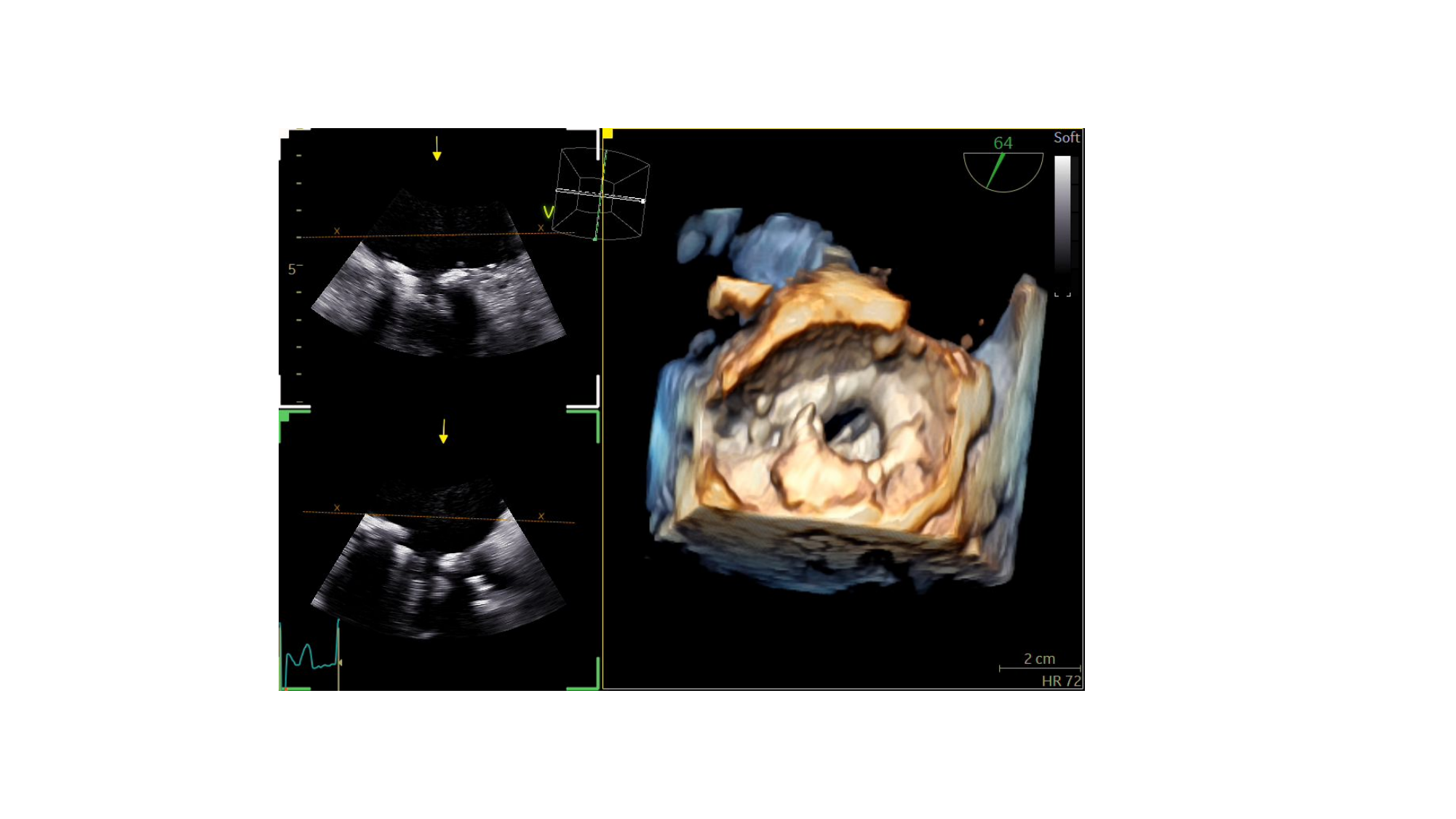

## Slide 4
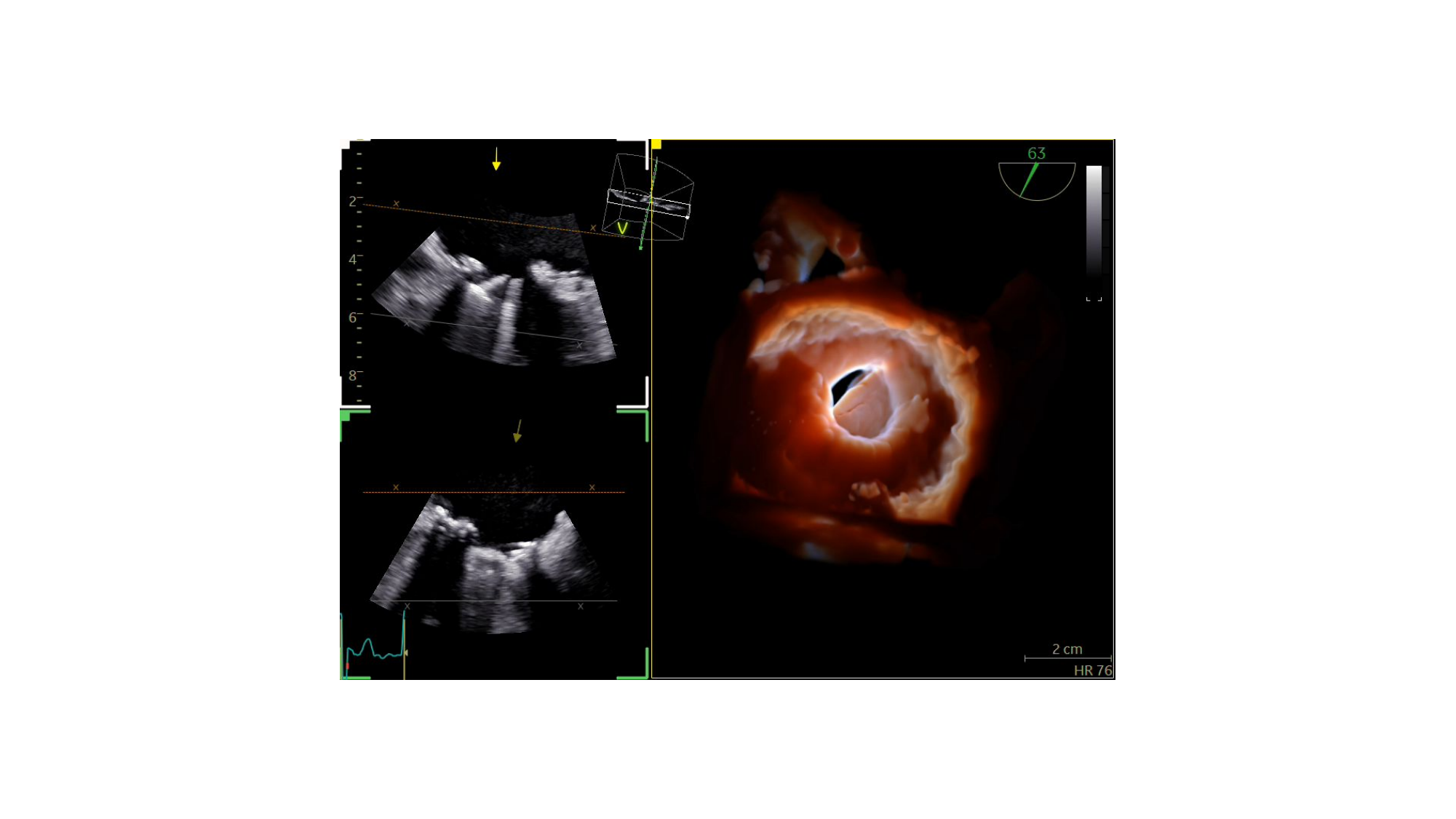

Supplement: Supplementary file 1 [file jcm-13-06450-s001.zip › VIDEO S4 METALLIC MV CASE.pptx]
