# Supplementary material for: Initial Experience with the 4D Mini-TEE Probe in the Adult Population
Source: J Clin Med. 2024 Oct 28;13(21):6450. doi: 10.3390/jcm13216450 (PMC11546711; doi:10.3390/jcm13216450)
Supplement: Supplementary file 1 [file jcm-13-06450-s001.zip › VIDEO S5 AOV CASE.pptx]

## Slide 1
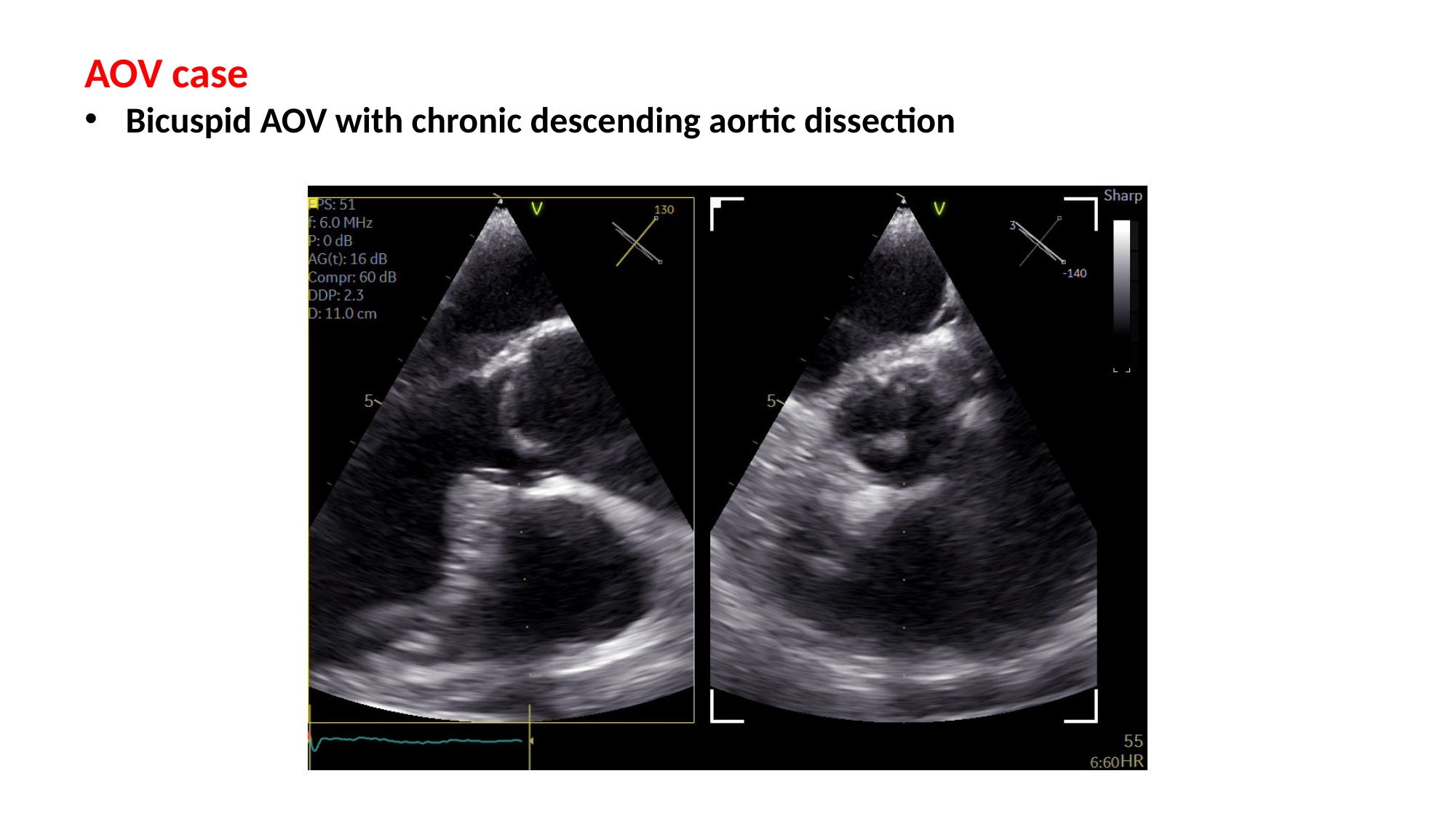

AOV case
Bicuspid AOV with chronic descending aortic dissection

## Slide 2
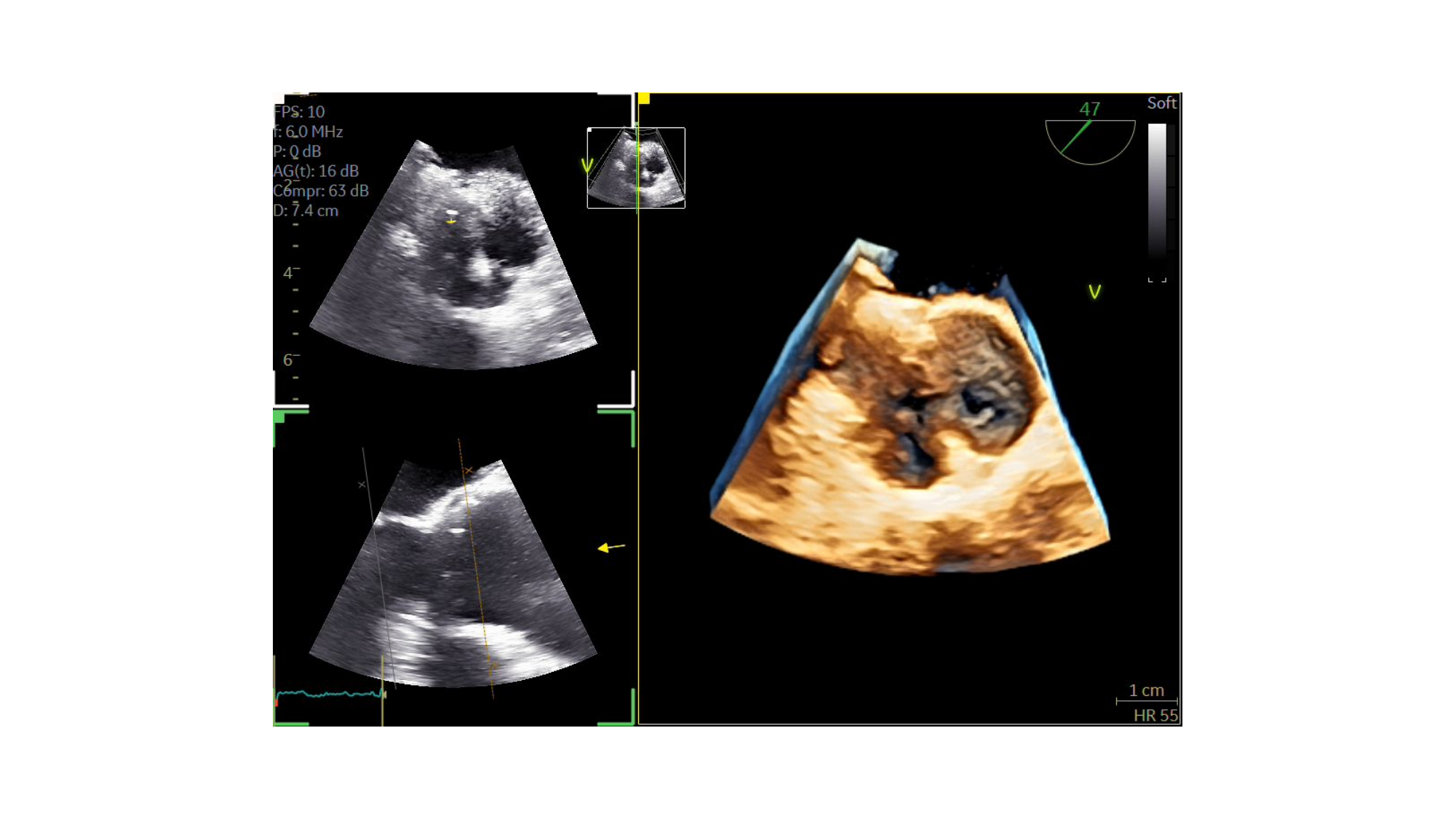

## Slide 3
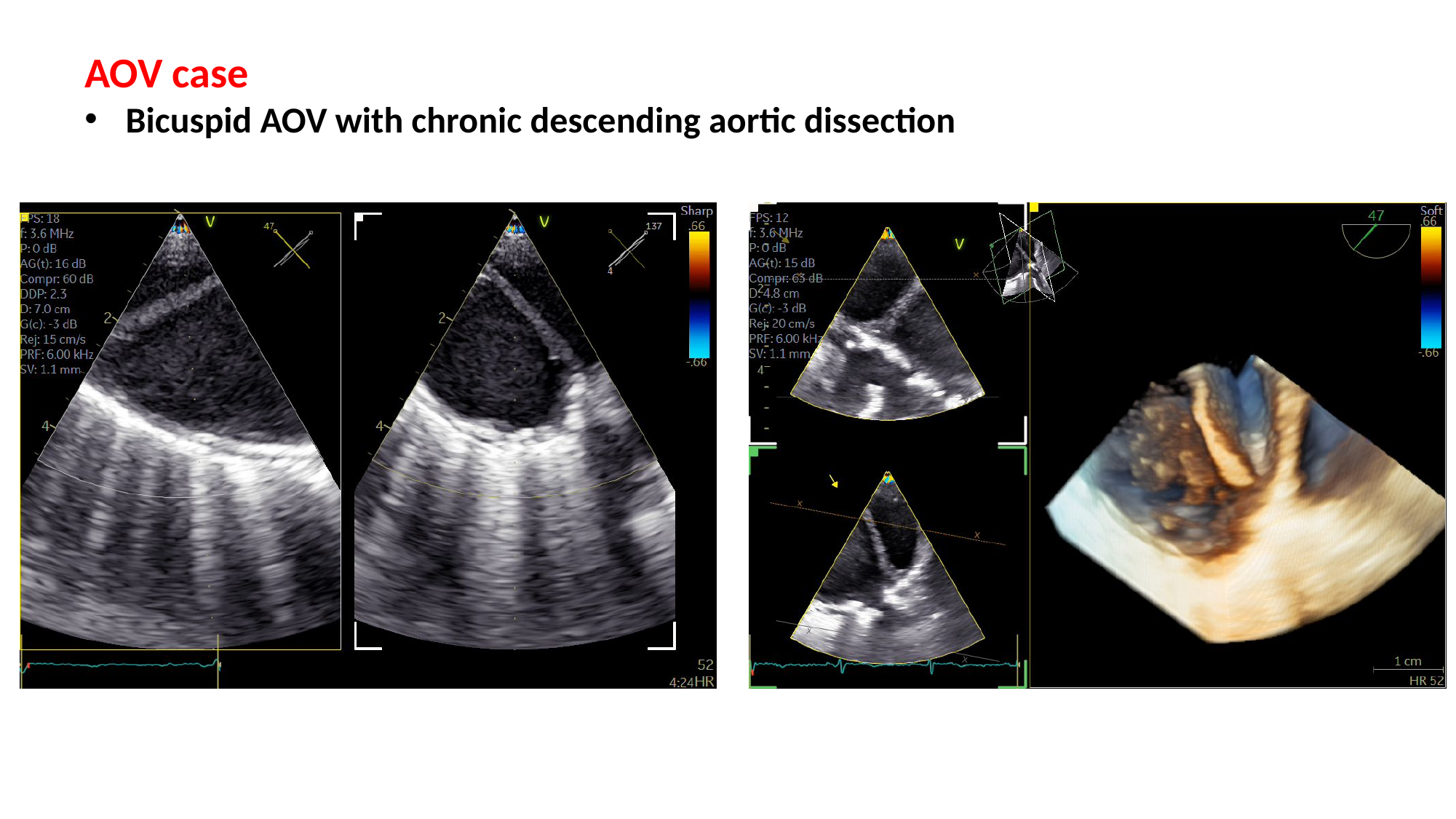

AOV case
Bicuspid AOV with chronic descending aortic dissection
