# Supplementary figures and images for: Initial Experience with the 4D Mini-TEE Probe in the Adult Population
Source: J Clin Med. 2024 Oct 28;13(21):6450. doi: 10.3390/jcm13216450 (PMC11546711; doi:10.3390/jcm13216450)

## Slide 1
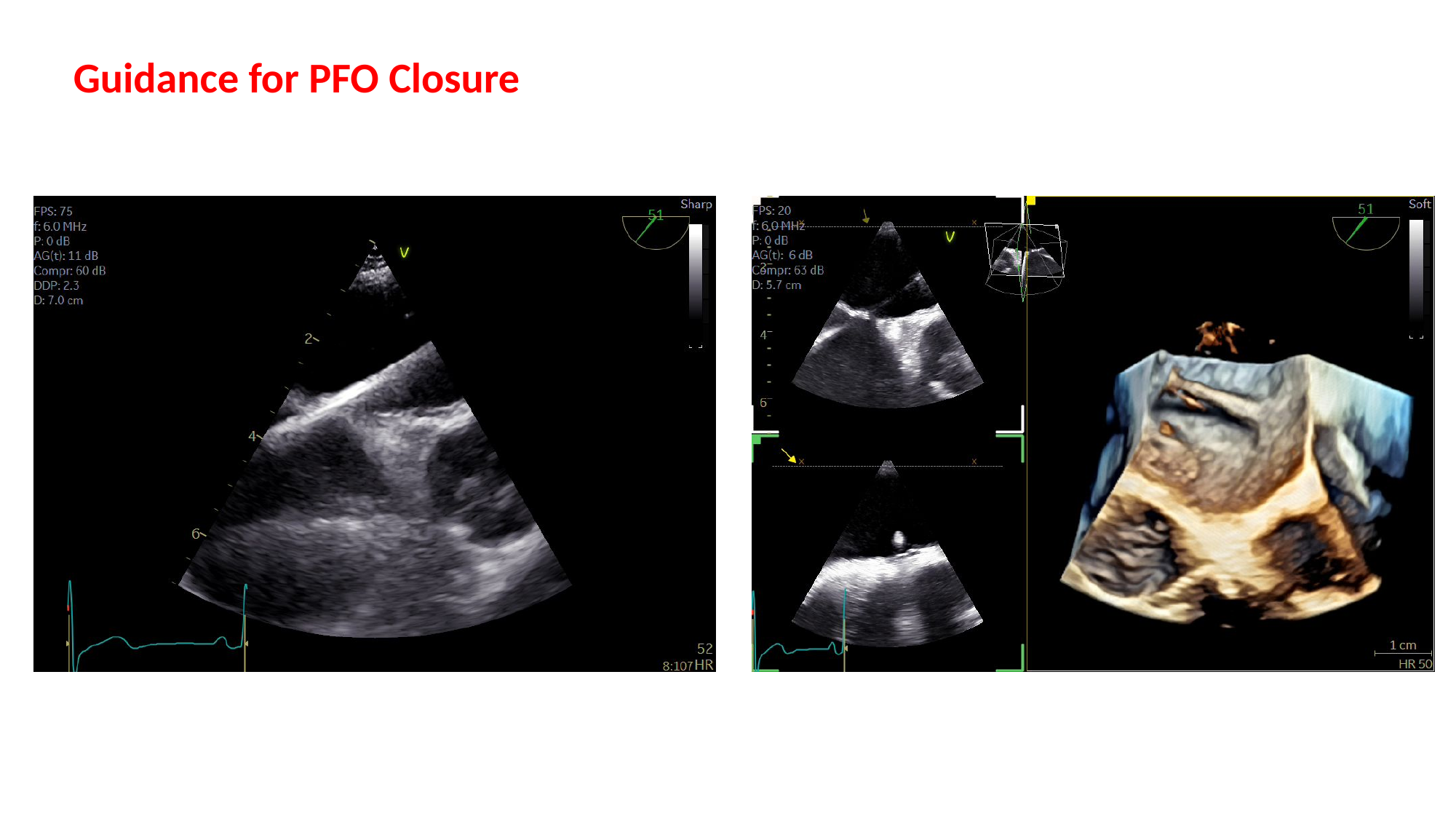

Guidance for PFO Closure

## Slide 2
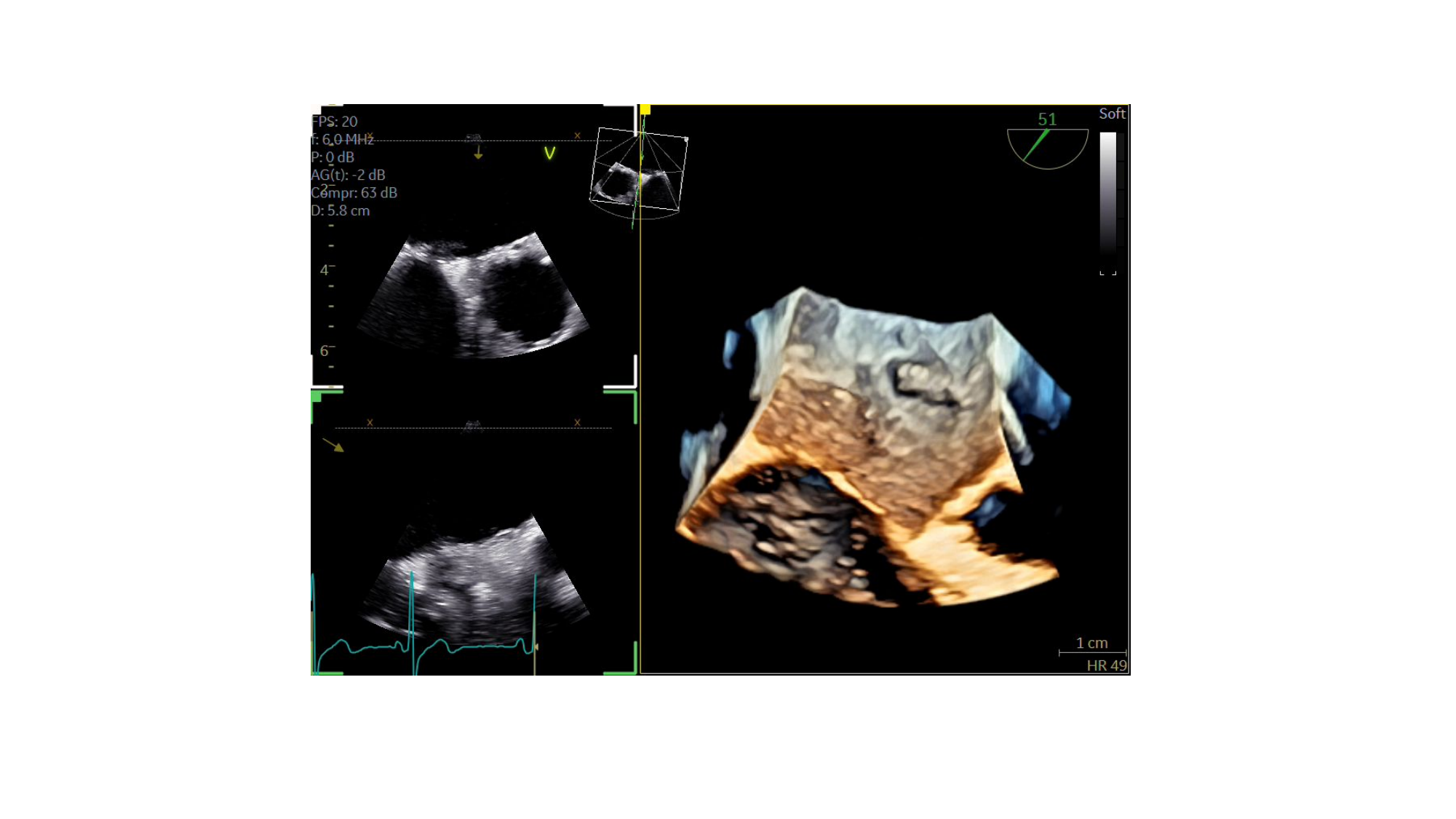

## Slide 3
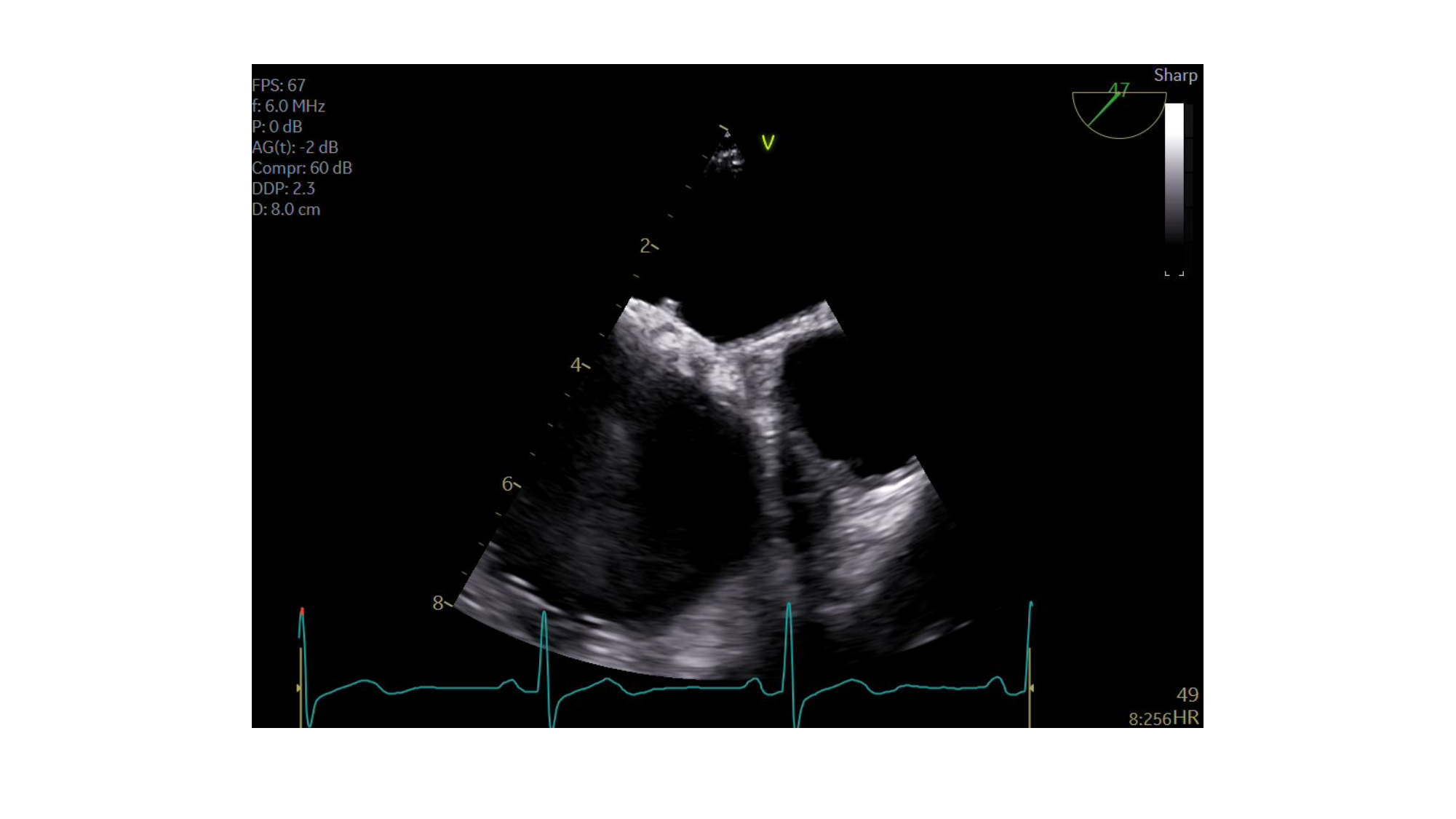

## Slide 4
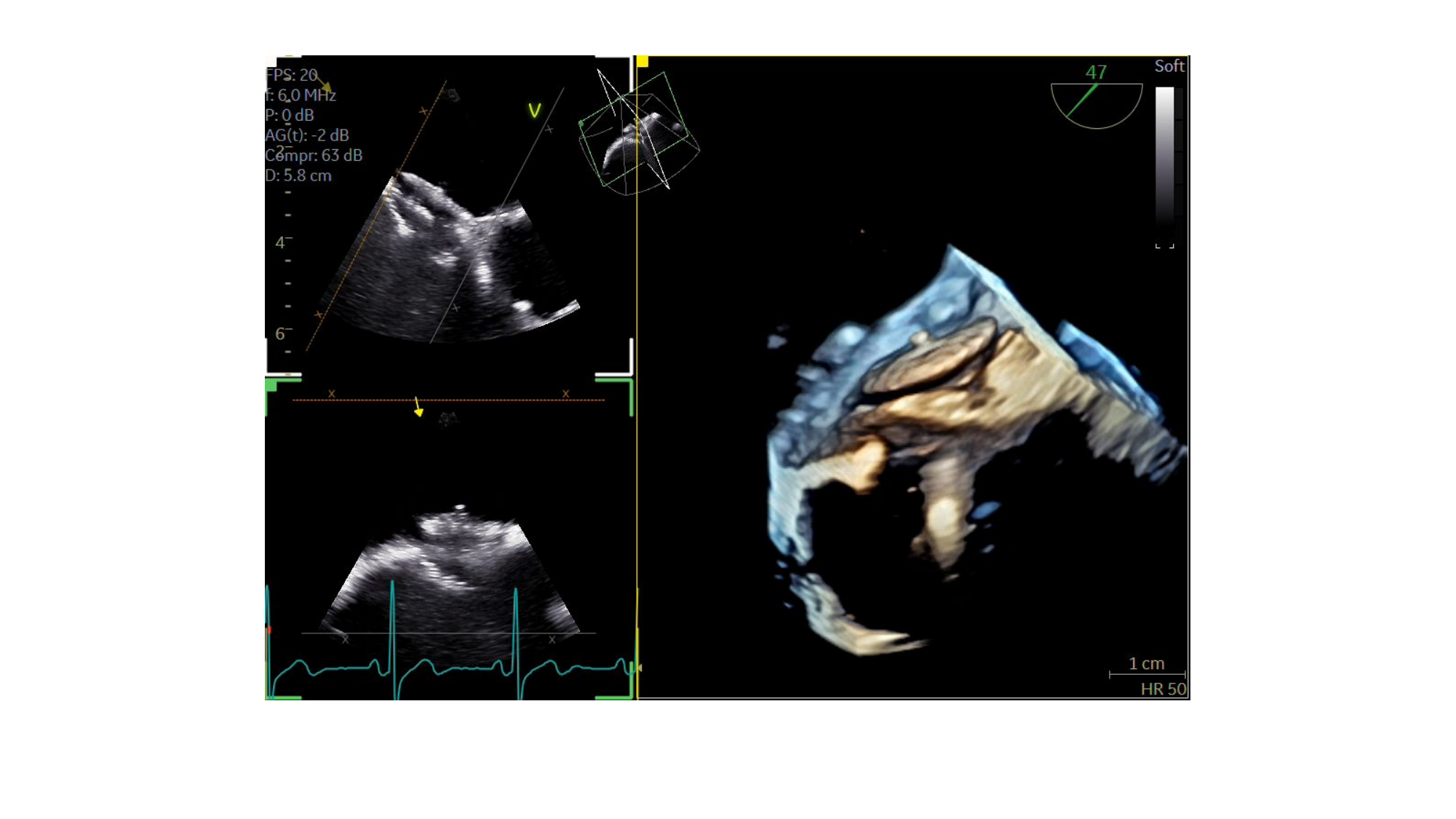

## Slide 5
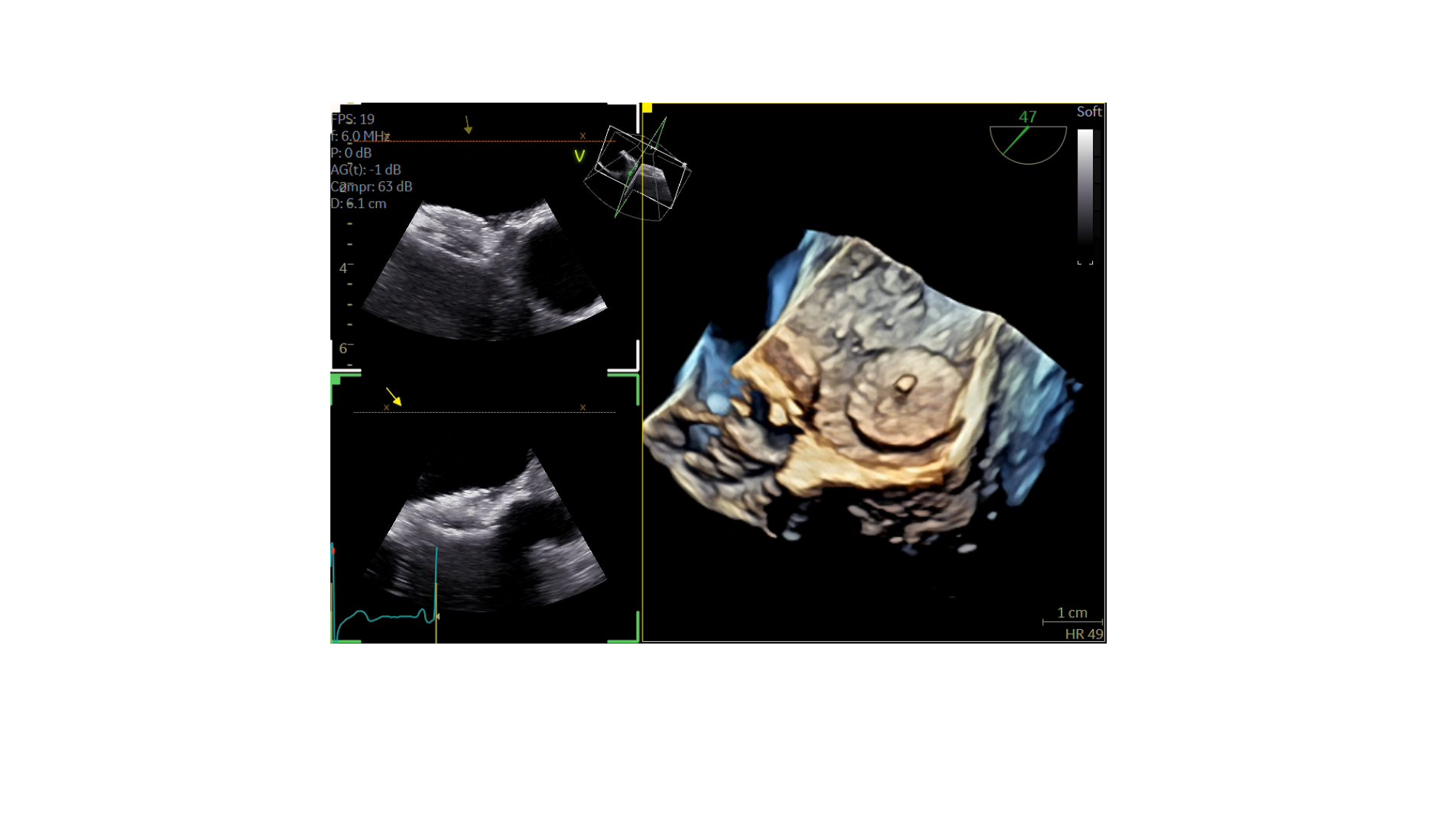

Supplement: Supplementary file 1 [file jcm-13-06450-s001.zip › VIDEO S2 PFO CLOSURE CASE.pptx]

## Slide 1
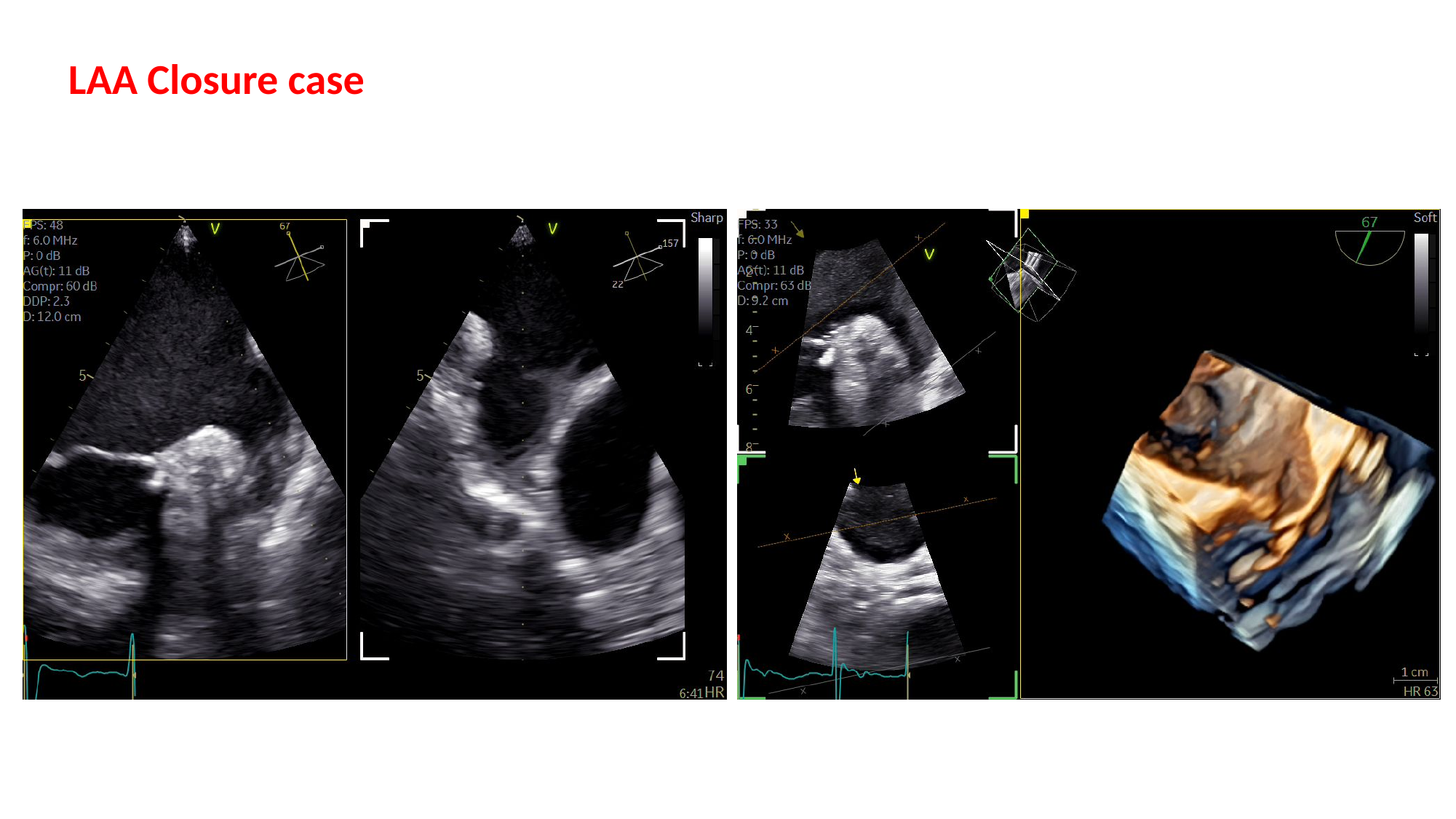

LAA Closure case

## Slide 2
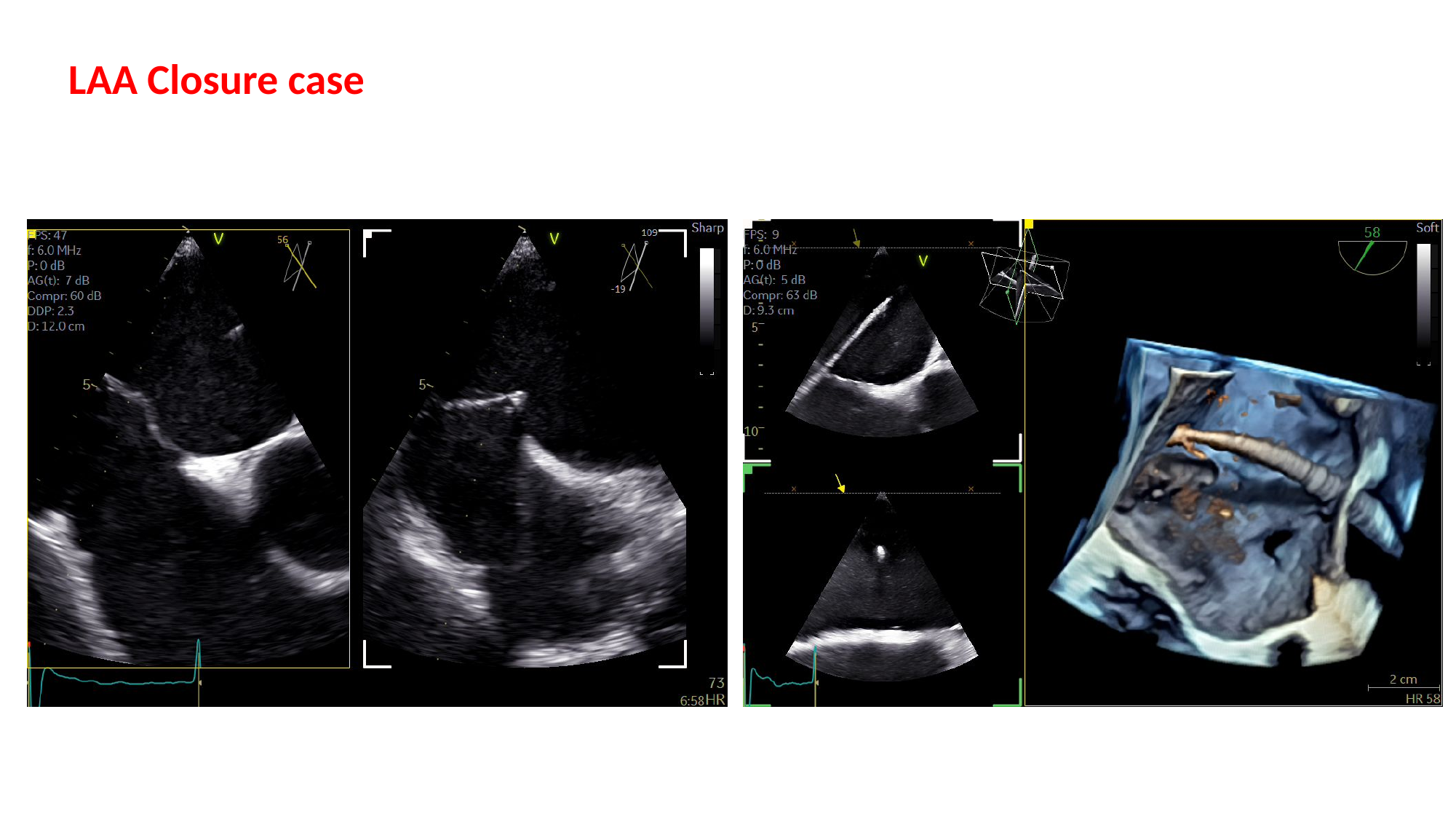

LAA Closure case

## Slide 3
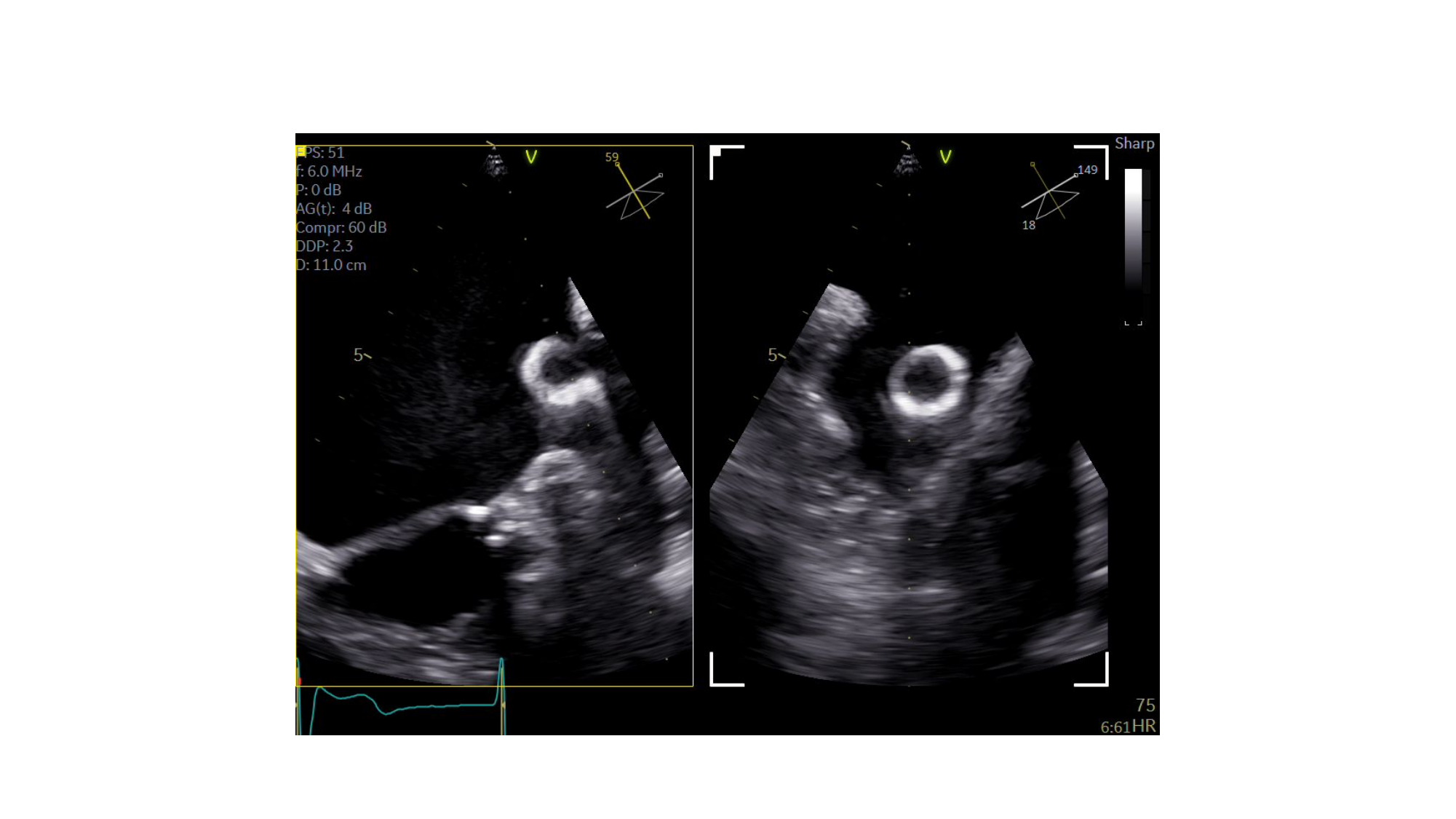

## Slide 4
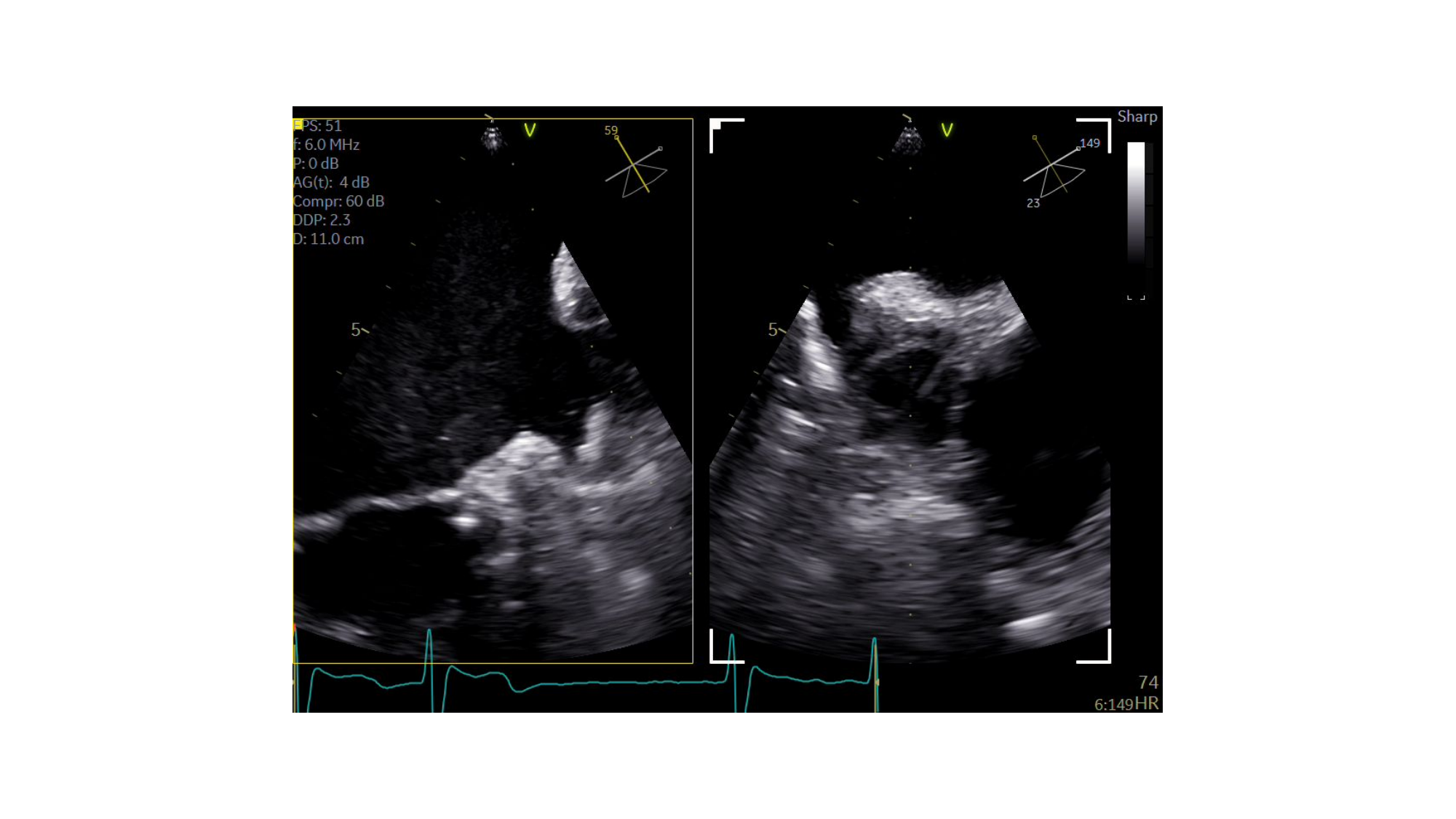

## Slide 5
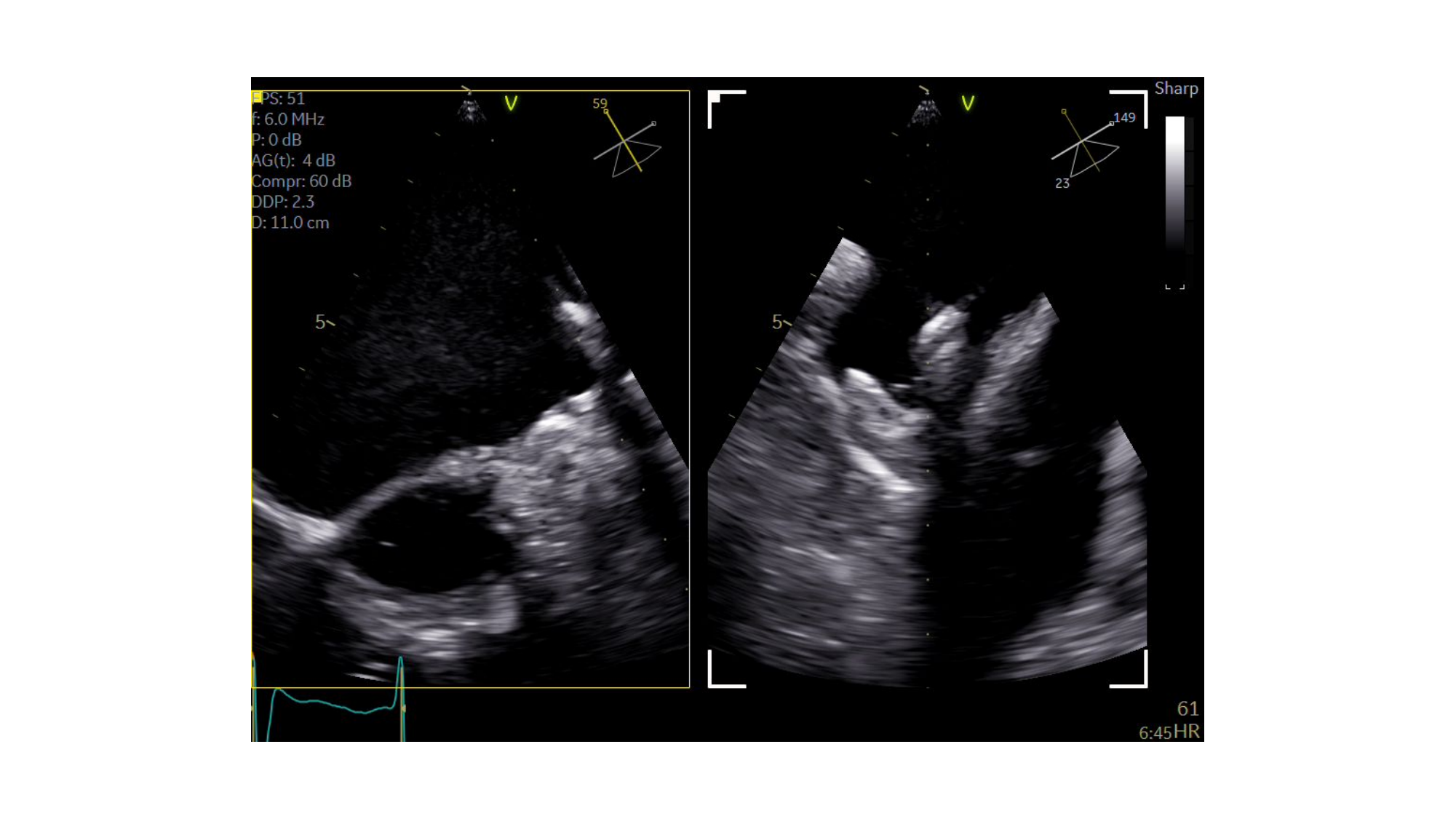

## Slide 6
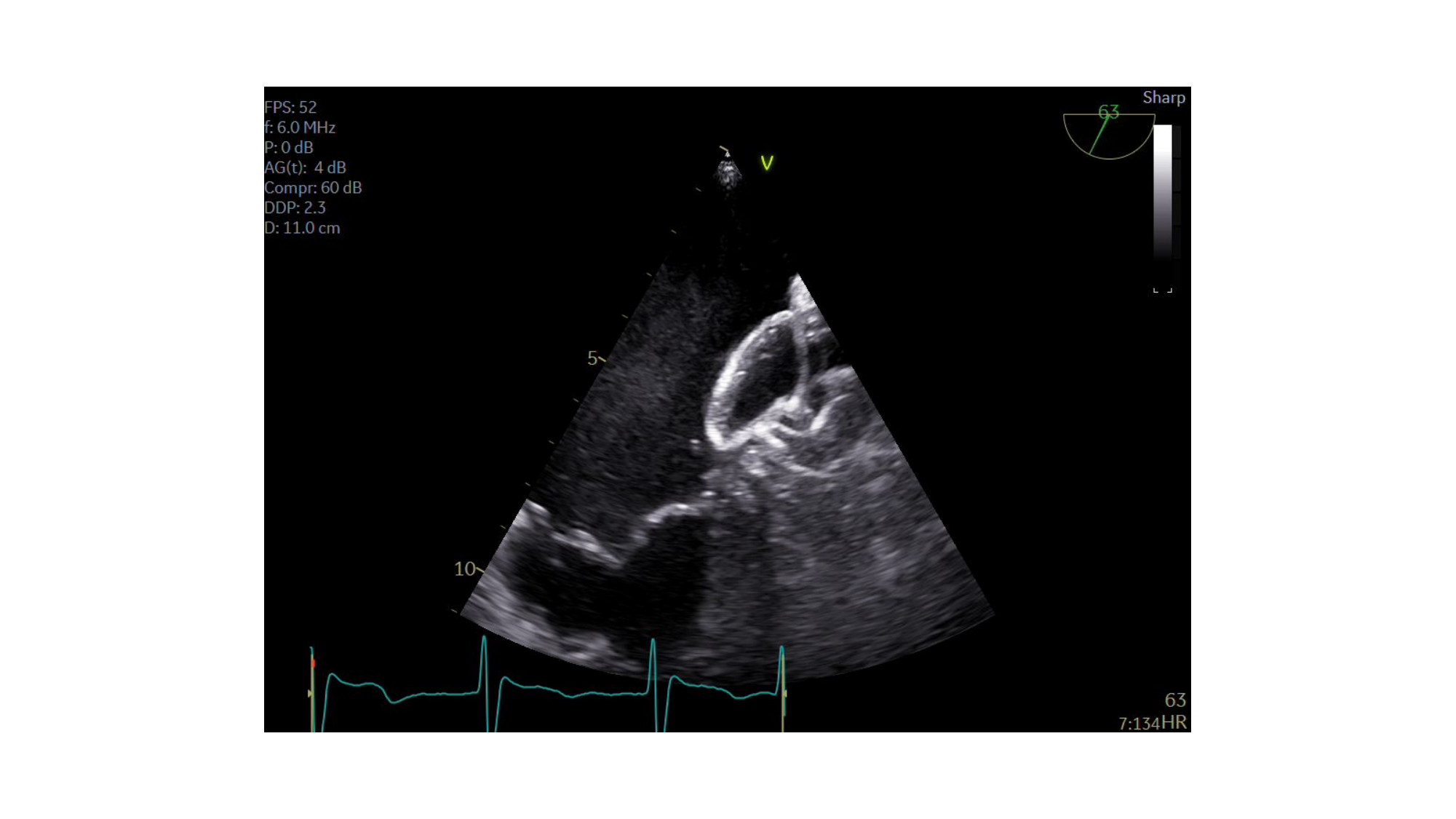

## Slide 7
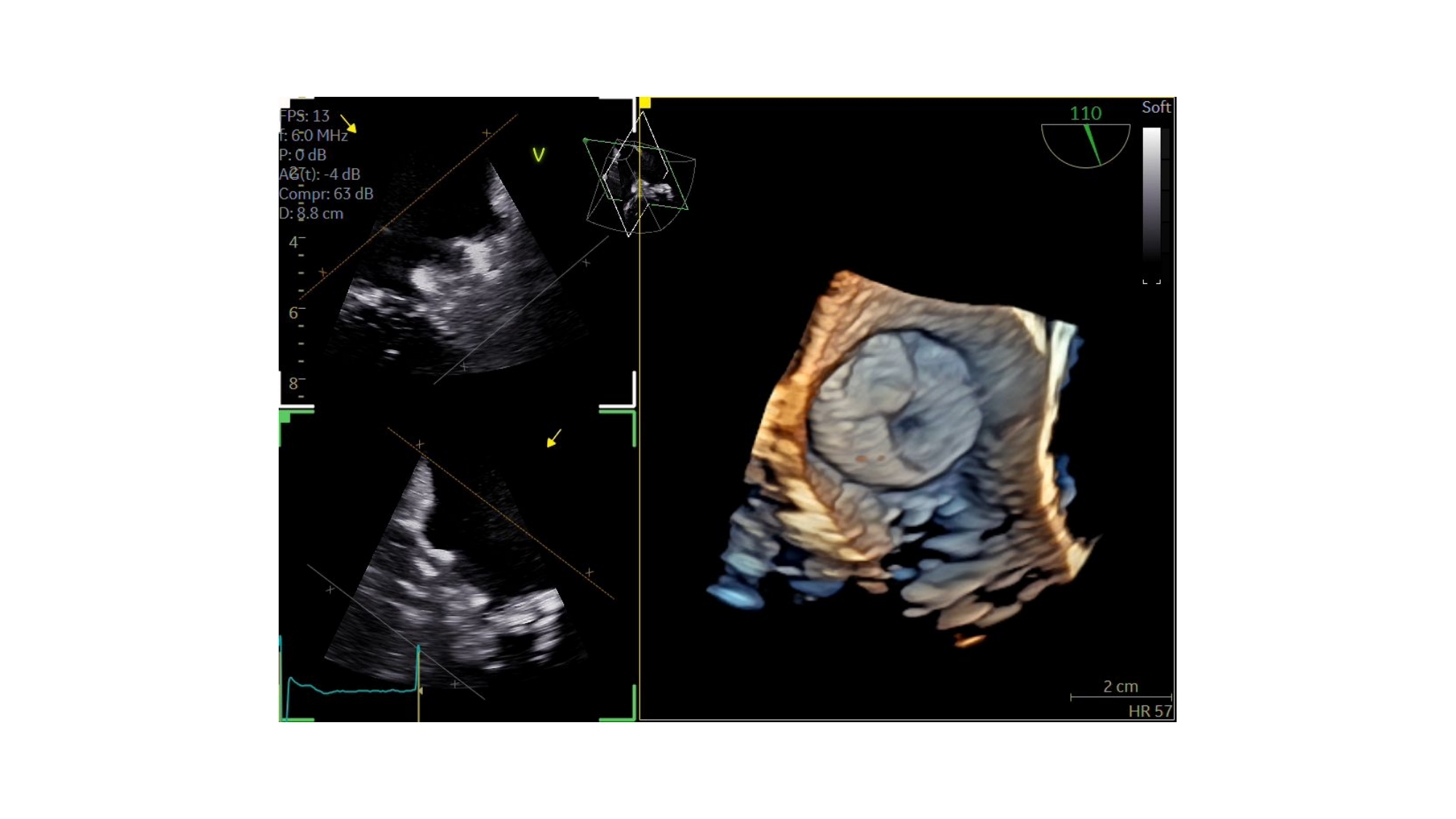

Supplement: Supplementary file 1 [file jcm-13-06450-s001.zip › VIDEO S3 LAA CLOSURE CASE.pptx]
